# Genetic and transcriptional dissection of resistance to *Claviceps purpurea* in the durum wheat cultivar Greenshank

**DOI:** 10.1007/s00122-020-03561-9

**Published:** 2020-02-14

**Authors:** Anna Gordon, Curt McCartney, Ron E. Knox, Nelzo Ereful, Colin W. Hiebert, David J. Konkin, Ya-Chih Hsueh, Vijai Bhadauria, Mara Sgroi, Donal M. O’Sullivan, Caroline Hadley, Lesley A. Boyd, Jim G. Menzies

**Affiliations:** 1 NIAB, 93 Lawrence Weaver Road, Cambridge, CB3 0LE UK; 2 Morden Research and Development Centre, Agriculture and Agri-Food Canada, Morden, MB R6M 1Y5 Canada; 3 Swift Current Research and Development Centre, Agriculture and Agri-Food Canada, Swift Current, SK Canada; 4 Aquatic and Crop Resource Development, National Research Council Canada, Saskatoon, SK Canada; 5 Department of Plant Sciences, University of Cambridge, Downing Street, Cambridge, CB2 3EA UK; 6 School of Agriculture, Policy and Development, University of Reading, Whiteknights, Reading, RG6 6AR UK

## Abstract

**Key message:**

Four QTL for ergot resistance (causal pathogen *Claviceps purpurea*) have been identified in the durum wheat cultivar Greenshank.

**Abstract:**

*Claviceps purpurea* is a pathogen of grasses that infects flowers, replacing the seed with an ergot sclerotium. Ergot presents a significant problem to rye, barley and wheat, in particular hybrid seed production systems. In addition, there is evidence that the highly toxic alkaloids that accumulate within sclerotia can cross-contaminate otherwise healthy grain. Host resistance to *C. purpurea* is rare, few resistance loci having been identified. In this study, four ergot resistance loci are located on chromosomes 1B, 2A, 5A and 5B in the durum wheat cv. Greenshank. Ergot resistance was assessed through analysis of phenotypes associated with *C. purpurea* infection, namely the number of inoculated flowers that produced sclerotia, or resulted in ovary death but no sclerotia, the levels of honeydew produced, total sclerotia weight and average sclerotia weight and size per spike. Ergot testing was undertaken in Canada and the UK. A major effect QTL, *QCp.aafc.DH*-*2A,* was detected in both the Canadian and UK experiments and had a significant effect on honeydew production levels. *QCp.aafc.DH*-*5B* had the biggest influence on total sclerotia weight per spike. *QCp.aafc.DH*-*1B* was only detected in the Canadian experiments and *QCp.aafc.DH*-*5A* in the UK experiment. An RNASeq analysis, undertaken to identify wheat differentially expressed genes associated with different combinations of the four ergot resistance QTL, revealed a disproportionate number of DEGs locating to the *QCp.aafc.DH*-*1B*, *QCp.aafc.DH*-*2A* and *QCp.aafc.DH*-*5B* QTL intervals.

**Electronic supplementary material:**

The online version of this article (10.1007/s00122-020-03561-9) contains supplementary material, which is available to authorized users.

## Introduction

**Fig. 1:**
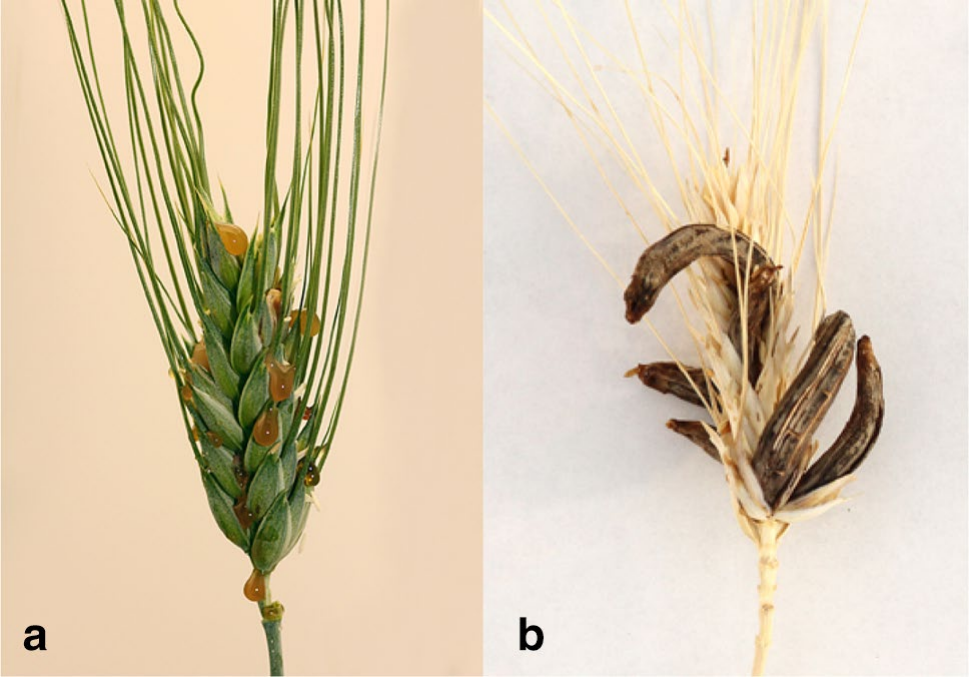
*Claviceps purpurea* infection symptoms on durum wheat. **a** Honeydew; infected flowers exude a mixture of *C. purpurea* conidiospores and plant sap. **b** Ergot sclerotia; *C. purpurea* overwintering structures

Ergot, caused by the fungal pathogen *Claviceps purpurea* (Fr.) Tul. (*Cp*), is a disease of cereals and grasses that infects female flowers at anthesis (Fig. 1; Menzies and Turkington 2015). Although commonly associated with open-flower pollinating species such as rye, ergot is also problematic for hexaploid, bread wheat (*Triticum aestivum* L.), and tetraploid, durum wheat *Triticum turgidum* L. subsp. *durum* (Desf.) Husn. Ergot is a significant problem for hybrid production systems for rye, barley and wheat production systems, where male sterility is induced to allow cross-pollination for F_1_ hybrid seed formation (Mantle and Swan 1995; Miedaner et al. 2010).

*Cp* spores germinate on mature stigma hairs and grow down the style towards the ovule. Microscopic studies suggest that the fungus does not grow beyond the rachis at the base of the ovary, but proliferates in the ovule tissues, occupying the area where a seed would normally develop (Haarmann et al. 2009). A mass of highly branched fungal hyphae, referred to as sphacelia, fills the ovule space. During this stage of infection, the fungus produces abundant asexual conidia suspended in a sugary sap that is exuded from the infected flower as honeydew (Fig. 1a). These conidia can be transported to new, uninfected flowers by rain splash and/or insects, resulting in new infections. Finally, around 4–6 weeks after infection, an ergot sclerotium, the fungal overwintering structure (Fig. 1b), is formed in place of a seed.

Ergot sclerotia are highly toxic to humans and animals due to a range of toxic alkaloids, commonly known as ergot alkaloids, which accumulate in the sclerotia (Shelby 1999; Beuerle et al. 2012). Ergot alkaloids have been deemed responsible for ergotism, known during the Middle Ages as St Anthony’s Fire. Symptoms include gangrenous extremities, convulsions, psychosis and can eventually lead to death. Outbreaks were especially prevalent in Europe during the Middle Ages due to the high proportion of rye and other cereals in the human diet (de Costa 2002).

Sclerotia are removed from grain by standard cleaning methods: colour sorting and gravity tables, with more rigorous scouring techniques being employed for rye (Beuerle et al. 2012; Byrd et al. 2017; MacDonald et al. 2017). However, when sclerotia are of a similar size to the cereal seed they are more challenging to separate. Wheat contaminated with sclerotia is downgraded at the elevator, or rejected at the mill, resulting in a financial loss to the farmer. Recent findings also suggest that alkaloid residues can find their way onto otherwise “healthy” grain, either during harvest and transportation, by physical contact with whole or damaged sclerotia, or via the spike, as a result of alkaloid transfer from infected flowers (Gordon et al. 2019).

Despite the importance of this disease, few sources of ergot resistance have been identified. Platford and Bernier (1970) first reported resistance in wheat to ergot, the resistance affecting the frequency and size of sclerotia and the amount of honeydew produced (Platford and Bernier 1976). They observed resistance in both hexaploid and tetraploid wheat, with the hexaploid cv. Kenya Farmer and the tetraploid cv. Carleton having the greatest resistance. Using cytogenetic analysis Platford et al. (1977) located resistance to ergot on chromosome 6B in Kenya Farmer and on chromosomes 1B, 3B, 4B and 5B in Carleton. Two ergot resistance QTL have been identified in the hexaploid wheat cv. Robigus, located on chromosomes 2A and 4B, and two from cv. Solstice on chromosomes 4D and 6A (Gordon et al. 2015), while in cv. Carberry and AC Cadillac, ergot resistance QTL were found on chromosomes 2B, 5A and 6A and chromosomes 2A, 3D, 6B and 7B, respectively (Berraies et al. 2019). Ergot resistance was identified in the CIMMYT durum wheat cv. Greenshank that reduced the number and size of sclerotia, as well as the amount of honeydew produced (Menzies 2004; Menzies et al. 2017).

The objectives of this study were to identify the genetic loci underlying ergot resistance in the cv. Greenshank using a series of phenotypes associated with *Cp* infection. Phenotypes included the success of *Cp* infection, levels of honeydew (HD) produced, total sclerotia weight per spike (TW) and average sclerotia weight (SW) and sclerotia size (SS) per spike. Experiments to determine ergot resistance were undertaken in Canada and the UK using a range of *Cp* isolates and led to the identification of four QTL contributing to ergot resistance. To investigate the molecular response conferred by these QTL, differential gene expression between DH lines carrying different combinations of these ergot resistance QTL was examined using RNA-Seq. Differentially expressed genes (DEGs) were mapped relative to each QTL to identify those DEGs associated with each QTL interval.

## Materials and methods

### Plant material and mapping population development

The cv. Greenshank (durum wheat line 9260B-173A), an entry from the CIMMYT 23rd International Durum Screening Nursery (CIMMYT accession number DW7588; http://wheatpedigree.net/sort/show/22773), was used in this study. AC Avonlea was developed at the Swift Current Research and Development Centre, Agriculture and Agri-Food, Canada. The average number of sclerotia per spike produced on Greenshank was 1.7, compared to 13.4 on AC Avonlea. Greenshank produced sclerotia with an average sclerotia size (SS) rating of 1.3, compared to 2.4 on AC Avonlea (on the Canadian three point scale, Supplementary file S1). For honeydew (HD), Greenshank was rated as 1.1 and AC Avonlea as 4.0 (on a four-point scale; Supplementary file S1; Menzies 2004).

Greenshank was crossed with AC Avonlea to produce a recombinant inbred line (RIL) population, using Greenshank as the female parent. Sixty RILs were produced by single seed descent to the F_5_ generation. An individual RIL, Greenshank_RIL3, was selected that conferred strong ergot resistance phenotypes (HD score of 1.0, TW = 1.9 mg, SW = 2.4 mg, SS = 0.42 and %Inf. = 4.0%) and the QTL identified in the RIL population. Greenshank_RIL3 was backcrossed to the susceptible cv. AC Avonlea to generate a doubled haploid (DH) population. The RIL was used as the female parent to make F_1_ seed, and a DH population of 132 lines was generated using the maize pollination procedure (Humphreys and Knox 2015).

### Pathogen inoculations and assessment of ergot resistance

Ergot resistance experiments were undertaken on the RIL population in Canada, while experiments on the DH population were undertaken in both Canada and the UK. In Canada, six *Cp* isolates, originating from Manitoba, Canada (Table 1), were mixed in equal concentrations in distilled water with one drop of Tween 20 (polyethylene glycol sorbitan monolaurate) per litre, making a final concentration of 10^4^ conidia per ml (Menzies 2004). In the UK, a single Canadian *Cp* isolate, EI4, was used (Table 1), and the *Cp* inoculum was prepared as described by Gordon et al. (2015). Fresh conidia were collected as honeydew and diluted in deionised water to a concentration 10^5^ conidia per ml. Flowers were inoculated using a hypodermic syringe.

**Table 1:** *Claviceps purpurea* isolates used in ergot resistance screens

*Claviceps purpurea* isolates	Original host and location of origin	Year of collection
EI1	Wheat (*T. aestivum*); Glenlea, Manitoba, Canada	1996
EI2	Wheat (*T. aestivum*); Glenlea, Manitoba, Canada	1996
EI3	Triticale (× Triticosecale); Glenlea, Manitoba, Canada	1996
EI4	Ergot from seed-cleaning plant; Oak River, Manitoba, Canada	1996
EI5	Ergot from seed-cleaning plant; Oak River, Manitoba, Canada	1996
EI6	Ergot from seed-cleaning plant; Oak River, Manitoba, Canada	1996

In Canada, plants were grown in temperature-controlled cabinets at 17–22 °C day/16 °C night, with a 15 h light/9 h dark cycle. The RIL population was grown as three plants per pot, with eight pots per line. Flowers on the three primary tillers of each plant were inoculated. The DH population was grown as two plants per pot, with three pots per line: the two primary tillers of each plant being inoculated. Twenty flowers were inoculated on each spike just before anthesis, with a minimum of ten spikes per line being inoculated. In the UK, the DH population was grown in temperature-controlled cabinets at 18 °C day/13 °C night, with a 16 h light/8 h dark cycle. Sixteen plants were grown per line (four plants per pot) with the primary spike of each plant being inoculated with *Cp*.

Approximately 14 days after inoculation (dai), each spike was assessed for honeydew production on a scale of 1–4, where 1 = no honeydew, 2 = honeydew confined within the glumes, 3 = honeydew exuding from the flowers in small drops and 4 = large drops of honeydew running down the spike (Supplementary file S1). Sclerotia were allowed to mature and collected approximately 40 dai. The total weight of sclerotia collected from each spike and the average weight and size of sclerotia per spike were recorded in both the Canadian and UK experiments. In the Canadian screens, sclerotia size was assessed on a 1–3 scale, while in the UK screens sclerotia size was scored on a 0–7 scale (Supplementary file S1). Percentage infection (%Inf) was measured as the number of inoculated flowers that formed sclerotia. Inoculated flowers where no sclerotia formed, but were left with a dried-out ovary, were scored as zero infection (%Zero).

Phenotypic data were analysed using Genstat 19th Edition (Rothamsted Experimental Station, Harpenden, UK). A SQRT or Log10 transformation was performed on data showing a non-normal distribution. The generalised linear model was used to obtain predicted means and *F*-statistics, along with accumulated variances. The predicted means of both the RIL and DH populations were used in the QTL analyses. Broad-sense heritability scores (H^2^) were calculated using the variance ratio outputs from the general linear regression (GLM) analysis in Genstat using the equation:

$${\text{H}}^{2} = \frac{{V_{\text{g}} }}{{V_{\text{g}} + V_{\text{e}} }}$$

where *V*_g_ is the genetic variance between lines and *V*_e_ is the error variance between replicates.

### Construction of the RIL and DH population genetic linkage maps

The RIL and DH populations were initially genotyped using DArT^®^ analysis [Wheat PstI (TaqI) v.3 chip; Akbari et al. 2006] and subsequently using the wheat 90 K Infinium iSelect array (Wang et al. 2014). Markers with DK prefixes were developed using Polymarker (Ramirez-Gonzalez et al. 2015) on variants found between honeydew-resistant DH lines A0262 and AM050 and three susceptible lines A0434-ER01, A0463 and AV013 that were obtained using the wheat-reduced exome capture (160318_Wheat_Tae_Red_EZ_HX1, Roche—NimbleGen). KASP markers were produced by LGC genomics (UK) based on SNPs identified within genes using the transcriptome RNA-Seq data (Supplementary file S2; Semagn et al. 2014). DNA was extracted according to the Triticarte protocol (Akbari et al. 2006).

Markers were placed into preliminary linkage bins using the BIN module in QTL IciMapping version 4.0.6.0 (Li et al. 2007, 2008) to identify co-segregating markers. A single marker, with the least missing data, was selected from each linkage bin, and maps were constructed for the RIL and DH populations using MapDisto (Lorieux, 2012). Linkage groups were identified based upon a minimum LOD score of 4.0 and a maximum recombination fraction of 0.2. Markers were ordered based upon a combination of automap, branch and bound, seriation, sum of adjacent recombination fraction and count.

### Identification of ergot resistance trait loci in cv. Greenshank

Quantitative trait loci (QTL) analysis was conducted using the BIP module of QTL IciMapping version 4.0.6.0 (Li et al. 2007, 2008). QTL analyses were undertaken with interval mapping (IM) and inclusive composite interval mapping (ICIM). Permutation tests (10,000 permutations) were used to determine an appropriate LOD threshold for QTL identification within each population. For the RIL population, LOD thresholds of 3.35 for IM-ADD (interval mapping looking for additive effect QTL) and 3.35 for ICIM-ADD (inclusive composite interval mapping looking for additive effect QTL) were used. For the DH population, a LOD threshold of 2.75 for IM-ADD and 2.75 for ICIM-ADD was used. QTL analysis statistics were calculated every 0.1 cM.

### RNA-Seq analysis of differentially expressed genes between Greenshank_RIL3 × AC Avonlea DH lines carrying different QTL combinations

Seventeen lines from the Greenshank_RIL3 × AC Avonlea DH population were selected that carried different combinations of the ergot resistance QTL (Supplementary file S3). Selections were made based on the parental alleles carried by the markers flanking each QTL interval, and the honeydew production levels and average scleroia-size phenotypes of each DH line. These seventeen DH lines were inoculated with the *Cp* isolate EI4. Ten–twelve infected ovules were dissected out of up to four spikes at 48 hpi and immediately placed into 0.5 ml RNA*later* (Sigma). After 24 h at room temperature, all samples were placed at − 80 °C until they could be processed together. Five to six infected ovules, up to two spikes, were combined to form two replicate samples of each DH line. Total RNA was extracted from each replicate set of ovules using the Plant RNEasy kit from Qiagen. RNA was treated with Turbo DNase (Thermo Fisher Scientific) according to the manufacturer’s instructions. RNA samples were sent on dry ice to the Earlham Institute, Norwich, UK, for RNA library construction using the TrueSeq 2.0 kit, and Illumina Hi-Seq 2.5 sequencing.

Single-end sequence reads were analysed for quality using FastQC (Andrews 2010). Overrepresented sequences, adapters and reads with low-quality base scores (at *Q* > 20) were removed from the RNASeq data. Reads were mapped and quantified using the quasi-mapping-based mode of Salmon (Patro et al. 2017), which both maps and quantifies the reads using the Svevo CDS as transcriptome reference (Maccaferri et al. 2019). A count matrix, containing the read counts of each library (as columns) with transcript names (as rows), was created. Pairwise differential gene expression analysis between ergot-resistant and susceptible groups (Supplementary file S3) was carried out applying quasi-likelihood F-test using edgeR (CRAN v3.4.2) (Robinson et al. 2010). A DGEList (Digital Gene Expression-List) object was made with the count matrix and a grouping factor as components. For the grouping factor, resistant lines were assigned as 1 and susceptible as 2. Poorly expressed isoforms were filtered out, the minimum requirement for isoform retention being at least one count per million (CPM) in at least three samples. Normalisation was performed with respect to library size using Trimmed Means of *M*-values (TMM), and the Cox–Reid method was used to estimate dispersions, both implemented in edgeR. Genes were considered as differentially expressed when they had a log2-fold change (L2FC) of > |1| and a false discovery rate of < 0.05. The predicted chromosomal locations and functional annotations assigned to DEGs are those provided by the Svevo genome sequence (Maccaferri et al. 2019). To identify the predicted physical interval of the four QTL, the sequences of all flanking markers (including co-segregating markers) from the LOD1 interval were compared with the Svevo genome using BLASTN.

## Results

### Phenotypic and genetic evaluation of ergot resistance in the Greenshank × AC Avonlea RIL population

**Fig. 2:**
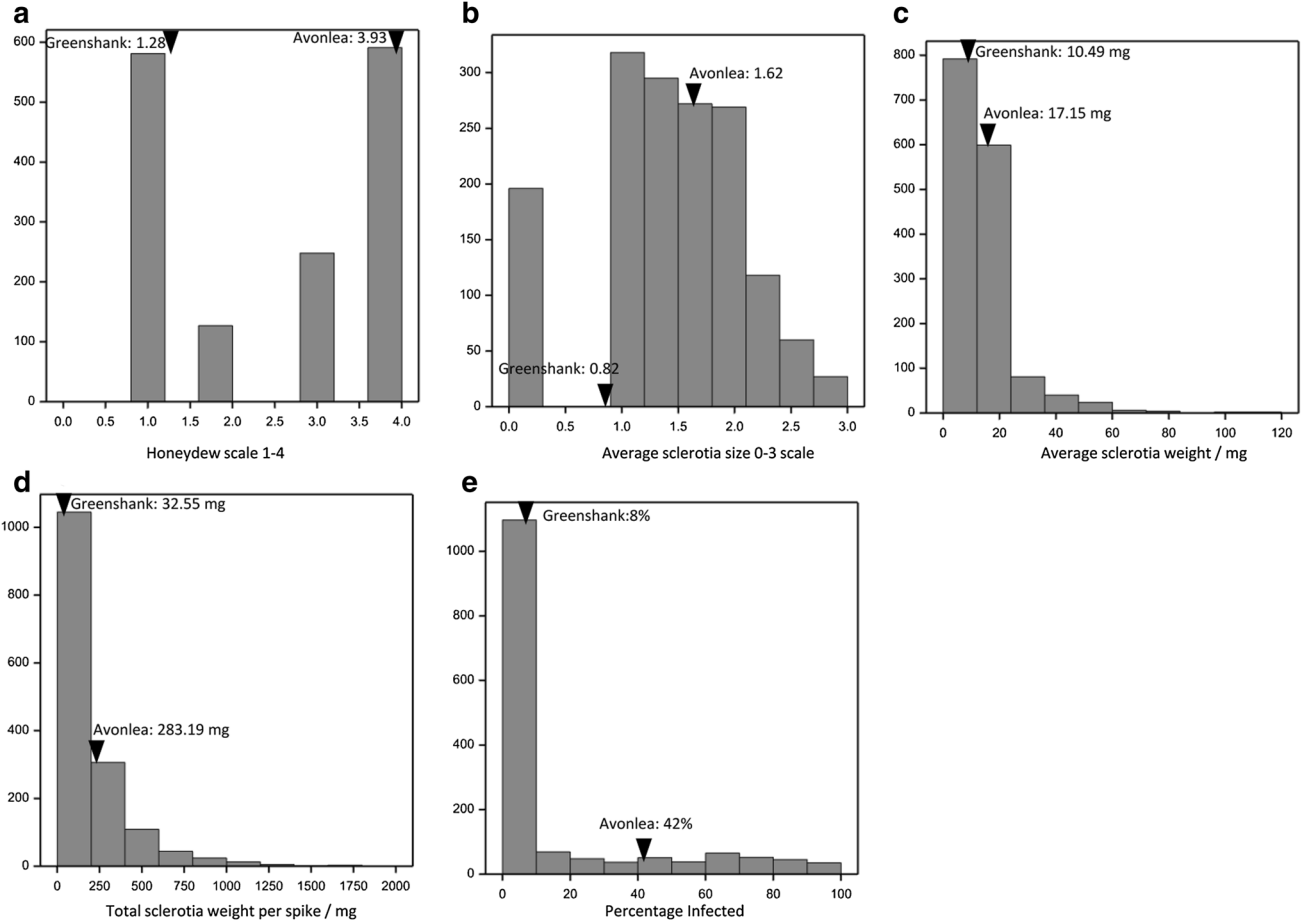
Phenotypic variation in five ergot resistance phenotypes measured in the RIL population derived from the cross-Greenshank × AC Avonlea. **a** Average honeydew production scores, measured on a 1–4 scale, **b** average sclerotia size per spike (SS), measured on a 1–3 scale, **c** average sclerotia weight per spike (SW), **d** total sclerotia weight per spike (TW) and **e** percentage infection, assessed as the percentage of *Claviceps purpurea* inoculated flowers that formed a sclerotia. The arrowheads indicate the phenotypic values of the parent cultivars. The *y*-axis depicts the number of data points per bar

Ergot resistance was initially assessed in a RIL population developed from the cross-Greenshank × AC Avonlea. Five phenotypes were assessed giving rise to the phenotypic distributions as shown in Fig. 2: honeydew production (HD), total sclerotia weight (TW), average sclerotia weight (SW), average sclerotia size (SS) per spike and percentage infection (%Inf). Quantitative variation, and possible transgressive segregation, was observed for average sclerotia size (Fig. 2b). Average sclerotia weight (Fig. 2c), total sclerotia weight (Fig. 2d), as well as percentage infection (Fig. 2e), all exhibited distributions skewed towards the resistant parent Greenshank. Honeydew production exhibited a more bimodal distribution: most RILs being either resistant like Greenshank, or susceptible like AC Avonlea (Fig. 2a).

A genetic linkage map was constructed for the RIL population (data not shown) consisting of 734 DArT, KASP and 90 k Illumina iSelect markers, across 48 linkage groups, with a total genetic distance of 1553 cM. QTL analysis of this RIL population identified QTL associated with honeydew production on chromosomes 1B (*QCp.aafc.RIL-1B)* and 2A (*QCp.aafc.RIL-2A)*, while QTL for total sclerotia weight per spike and percentage infection were detected on chromosome 2A (Table 2). A RIL (Greenshank_RIL3) was selected that conferred strong ergot resistance phenotypes (HD score of 1.0, TW of 1.9 mg, SW of 2.4 mg, SS score of 0.42 and %Inf value of 4.0%) and contained the QTL intervals on chromosomes 1B and 2A. This RIL was backcrossed to AC Avonlea to generate a larger, doubled haploid (DH) population.

**Table 2:** Ergot resistance QTL identified using inclusive composite interval mapping (ICIM) in the Greenshank × AC Avonlea recombinant inbred line (RIL) population

QTL	Trait^a^	LOD^b^	Chrom. location	Peak position/CM	Left marker	Right marker	% variance explained	Add	LOD1 left^c^	LOD1 right^c^
*QCp.aafc.RIL-1B*	HD_Ca_RIL	3.40	1B	72.8	Tdurum_contig77676_464	RAC875_c8878_232	4.2	0.316	71.35	74.35
*QCp.aafc.RIL-2A*	TW_Ca_RIL	5.50	2A	9.268	Ku_c28953_422	Ku_c10387_272	31.9	0.222	8.52	9.32
	HD_Ca_RIL	19.56		9.868	Kukri_c79633_88	Excalibur_rep_c107890_89	37.8	0.940	9.42	10.12
	%Inf_Ca_RIL	5.22		9.268	Ku_c10387_272	Ku_c28953_422	33.8	0.795	8.52	9.32

^a^Trait names refer to honeydew production levels (HD), total sclerotial weight (TW) per spike, and percentage of infected flowers (%Inf) that formed sclerotia

^b^LOD significance threshold for ICIM was 3.35

^c^LOD1 left and LOD1 right refer to the LOD1 confidence interval

### Phenotypic evaluation of ergot resistance in the Greenshank_RIL3 × AC Avonlea DH population

Greenshank_RIL3 was backcrossed to the ergot susceptible line AC Avonlea, and 132 DH lines were generated. This DH population was phenotyped in Canada using a mixture of six *Cp* isolates (Table 1), and in the UK using the Canadian *Cp* isolate EI4. In the UK screen, an additional ergot resistance trait was recorded: zero infection (%Zero), this was the percentage of inoculated flowers that did not produce an ergot sclerotia but left a dried-out ovary. The segregation patterns of the ergot resistance phenotypic traits in the DH population were very similar to those seen in the RIL population, except for the %Inf UK scores, which presented a broader distribution (Supplementary file S4).

High broad-sense heritability was seen for honeydew production with H^2^ values ranging from 0.95 to 0.76. In general, H^2^ values for total sclerotia weight per spike ranged from 0.87 to 0.79 and from 0.77 to 0.64 for average sclerotia weight and from 0.81 to 0.73 for average sclerotia size. For %Inf, H^2^ values ranged from 0.92 to 0.84. However, with the Canadian DH data sets the H^2^ values for these four phenotypes were 0.13, 0.13, 0.15 and 0.13, respectively. It is unclear why such low H^2^ values were observed with these data sets although this screen did apply a mixture of six *Cp* isolates, compared to the single isolate applied in the UK screen. Infection Zero, measured only in the UK DH population screens, had a H^2^ value of 0.90 (Supplementary file S5).

### Genetic evaluation of ergot resistance in the Greenshank_RIL3 × AC Avonlea DH population

A genetic linkage map was constructed for the DH population composed of 357 marker loci and 21 linkage groups, with a total genetic distance of 763 cM (Supplementary file S6). Despite the DH population being derived from a backcross between Greenshank_RIL3 × Avonlea, all 14 chromosomes, except 7A, were represented.

**Fig. 3:**
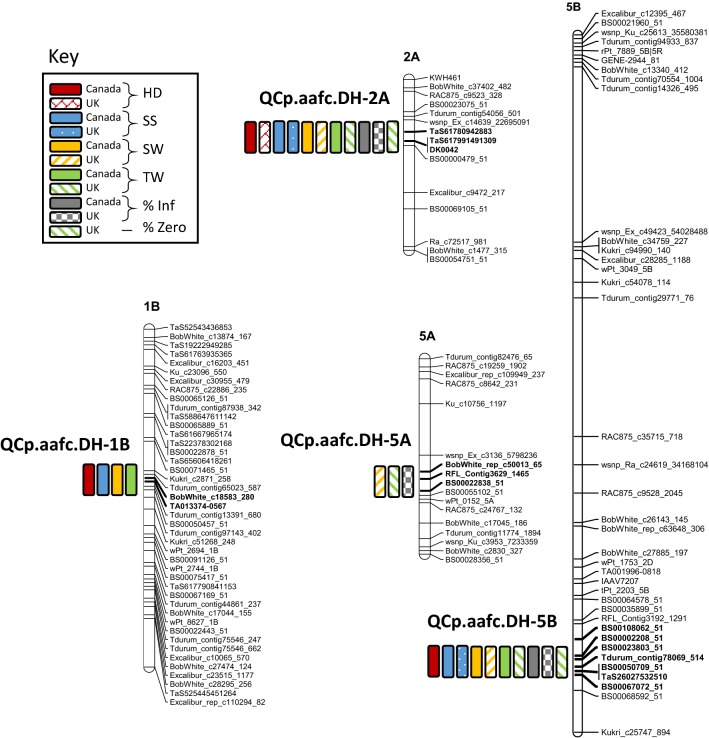
Ergot resistance QTL identified in the Greenshank_RIL3 × AC Avonlea DH population. Four QTL were identified using phenotypic data for HD—honeydew production level, TW—total sclerotia weight, SW—average sclerotia weight, SS—average sclerotia size per spike,  %Inf—the percentage of *Claviceps purpurea* inoculated flowers that developed a sclerotia, and  %Zero—the percentage of *C. purpurea* inoculated flowers that did not develop sclerotia, but were left with a dried-out ovary

Four QTL for ergot resistance were identified in the Greenshank_RIL3 × AC Avonlea DH population; *QCp.aafc.DH*-*1B*, *QCp.aafc.DH*-*2A, QCp.aafc.DH*-*5A* and *QCp.aafc.DH*-*5B.* These QTL were all derived from Greenshank (Table 3 and Fig. 3). All phenotypic data sets gave rise to a significant QTL on chromosome 2A, *QCp.aafc.DH*-*2A. QCp.aafc.DH*-*2A* was prominent in both the Canadian and UK screens, although the QTL explained a larger percentage of the phenotypic variances in the UK screen. *QCp.aafc.DH*-*2A* explained 90.9% of the genetic variance in honeydew levels in the UK data set and 71.9% in the Canadian data set. *QCp.aafc.DH*-*2A* was located in the same region on chromosome 2A as *QCp.aafc.RIL*-2A.

**Table 3:** Ergot resistance QTL identified using inclusive composite interval mapping (ICIM) in the Greenshank_RIL3 × AC Avonlea doubled haploid (DH) population

QTL	Trait name^a^	LOD^b^	Chrom.	Peak position/cM	Left marker	Right marker	% variance	Add	LOD1 left^c^	LOD1 right^c^
*QCp.aafc.DH-1B*	HD_Ca_DH	4.02	1B	16.6	BobWhite_c18583_280	TA013374-0567	2.9	0.1601	16.25	17.05
	SS_Ca_DH	3.80	1B	16.6	BobWhite_c18583_280	TA013374-0567	7.8	0.1319	16.25	17.05
	SW_Ca_DH	3.24	1B	16.6	BobWhite_c18583_280	TA013374-0567	5.7	0.1475	16.25	17.05
	TW_Ca_DH	3.33	1B	16.6	BobWhite_c18583_280	TA013374-0567	4.7	0.1933	16.25	17.05
*QCp.aafc.DH-2A*	HD_Ca_DH	42.41	2A	5.7	TaS61780942883	DK0042	71.9	0.7956	4.95	6.65
	HD_UK_DH	43.24	2A	5.7	TaS61780942883	DK0042	90.0	1.1612	4.75	6.65
	SS_Ca_DH	7.21	2A	5.7	TaS61780942883	DK0042	15.8	0.1876	4.35	6.75
	SS_UK_DH	10.41	2A	5.7	TaS61780942883	DK0042	40.8	0.4948	4.65	6.75
	SW_Ca_DH	10.86	2A	5.7	TaS61780942883	DK0042	22.1	0.2908	4.65	6.65
	SW_UK_DH	10.65	2A	5.7	TaS61780942883	DK0042	39.6	0.2939	4.65	6.75
	TW_Ca_DH	15.48	2A	5.7	TaS61780942883	DK0042	28.0	0.4706	4.75	6.65
	TW_UK_DH	14.49	2A	5.7	TaS61780942883	DK0042	46.0	0.4698	4.75	6.65
	%Inf_Ca_DH	17.17	2A	5.7	TaS61780942883	DK0042	33.9	1.1255	4.55	6.65
	%Inf_UK_DH	19.67	2A	5.7	TaS61780942883	DK0042	56.4	20.0739	4.75	6.65
	%Zero_UK_DH	17.95	2A	5.7	TaS61780942883	DK0042	61.4	− 2.2389	4.75	6.65
*QCp.aafc.DH-5A*	%Inf_UK_DH	5.07	5A	14.4	RFL_Contig3629_1465	BS00022838_51	8.8	7.9162	12.35	14.45
	SW_UK_DH	2.97	5A	12.6	BobWhite_rep_c50013_65	RFL_Contig3629_1465	8.8	0.1385	10.55	13.15
	TW_UK_DH	5.33	5A	13.2	RFL_Contig3629_1465	BS00022838_51	12.5	0.2444	12.25	14.45
*QCp.aafc.DH-5B*	HD_Ca_DH	9.04	5B	69.3	Tdurum_contig78069_514	BS00050709_51	7.2	0.2547	68.55	69.65
	SS_Ca_DH	11.81	5B	69.6	Tdurum_contig78069_514	BS00050709_51	28.6	0.255	69.25	69.65
	SS_UK_DH	4.72	5B	69.6	Tdurum_contig78069_514	BS00050709_51	15.6	0.3086	69.25	69.65
	SW_Ca_DH	14.52	5B	69.6	Tdurum_contig78069_514	BS00050709_51	32.1	0.3546	69.25	69.65
	SW_UK_DH	3.79	5B	69.6	Tdurum_contig78069_514	BS00050709_51	11.4	0.1593	69.25	69.65
	TW_Ca_DH	18.16	5B	69.6	Tdurum_contig78069_514	BS00050709_51	35.0	0.5322	69.25	69.65
	TW_UK_DH	3.95	5B	69.6	Tdurum_contig78069_514	BS00050709_51	8.9	0.2087	69.25	69.65
	%Inf_Ca_DH	16.41	5B	69.7	TaS26027532510	BS00067072_51	31.9	1.1027	69.65	70.15
	%Inf_UK_DH	2.74	5B	68.0	BS00002208_51	BS00023803_51	4.5	5.6374	66.25	68.35
	%Zero_UK_DH	2.72	5B	67.9	BS00108062_51	BS00002208_51	5.6	− 0.6764	66.25	68.35

^a^Trait names refer to honeydew production levels (HD), total sclerotia weight (TW), average sclerotia weight (SW) and average sclerotia size (SS) per spike, percentage of inoculated flowers producing a sclerotia (%Inf) and percentage of inoculated flowers (%Zero) with a dried-out ovary

^b^LOD significance threshold for ICIM was 2.75

^c^LOD1 left and LOD1 right refer to the positions of the LOD1 confidence interval

All phenotypic data sets, except UK honeydew levels, detected a QTL on chromosome 5B in the same location: *QCp.aafc.DH*-*5B*. *QCp.aafc.DH*-*5B* had a far bigger effect in the Canadian ergot resistance screen, where six *Cp* isolates were used, and contributed most to reducing total ergot sclerotia weight per spike. *QCp.aafc.DH*-*1B* was only detected in the Canadian ergot resistance screen, having a significant effect on honeydew production levels, total sclerotia weight and average sclerotia weight and size per spike. *QCp.aafc.DH*-*5A* was only detected in the UK screen, being significant for percentage infection, total and average sclerotia weight per spike.

Alignment of the iSelect SNP markers defining each QTL region on the durum wheat Svevo reference genome sequence (Maccaferri et al. 2019) allowed us to determine the physical size of each QTL interval. *QCp.aafc.DH*-*2A* spanned a 17.5 Mb region incorporating 277 high-confidence (HC) and 594 low-confidence (LC) predicted genes. *QCp.aafc.DH*-*1B* spanned an 82.6-Mb region containing 2796 genes (368 HC and 2428 LC genes), *QCp.aafc.DH-5A* a 1.86-Mb region incorporating 87 genes (25 HC and 52 LC genes) and *QCp.aafc.DH-5B* a 373-Mb region incorporating 12,209 genes (1855 HC and 10354 LC genes).

### RNASeq analysis of differential wheat gene expression between Greenshank_RIL3 × AC Avonlea DH lines carrying different QTL combinations

An RNASeq analysis was undertaken to determine how gene expression differed in infected wheat ovaries relative to the QTL present. Seventeen DH lines, carrying different combinations of the four ergot resistance QTL, were selected (Supplementary file S7). Lines having a honeydew score of < 1.5 and an average ergot sclerotia size of < 2 (UK sizing score) were deemed resistant. Lines selected as susceptible had a honeydew score > 3 and an average sclerotia size > 3. Seventeen DH lines were inoculated with a single *Cp* isolate, EI4 and RNA extracted from dissected ovaries 48 hai.

DH lines were grouped based on the ergot resistance QTL they carried, and pairwise comparisons made between groups, as described in Supplementary file S7, to identify DEGs associated with specific ergot resistance QTL combinations. The pairwise comparison of GR1 and GS1 identified 70 DEGs associated with the QTL on chromosome 2A. All ergot-resistant lines in GR1 contained the 2A QTL, either singularly, or in combination with the QTL on chromosomes 1B, 5A and 5B, while *QCp.aafc.DH*-*2A* was absent from the group GS1, which contained the other three ergot resistance QTL. Comparison of the group GR2, which possessed *QCp.aafc.DH*-*2A-* and *QCp.aafc.DH*-*5B*-resistant alleles from Greenshank_RIL3, with group GS2 (lines lacking the QTL *QCp.aafc.DH*-*2A* and *QCp.aafc.DH*-*5B*) returned 133 DEGs. The GR3 to GS3 pairwise comparison of lines specifically carrying *QCp.aafc.DH*-*2A* and *QCp.aafc.DH*-*1B* returned 202 DEGs. The pairwise comparison GR4 to GS4, comparing lines containing *QCp.aafc.DH*-*2A* and *QCp.aafc.DH*-*5A* to lines without these QTL, returned 27 DEGs. The GR5 to GS5 comparison was of lines with the QTL on chromosomes 1B, 2A and 5B (GR5) to a group of lines that were null for 1B, 2A and 5B, but containing 5A. This comparison returned 249 DEGs. A comparison of lines containing all four QTL vs null lines was not possible as only one DH line was null for all four QTL, providing insufficient statistical power. The results of these pairwise analyses are summarised in Supplementary file S8. The relationship between the DEGs found in each of the five pairwise comparisons can be summarised in the Venn diagram presented in Supplementary file S9. Full information on the DEGs can be found in Supplementary File S10.

**Fig. 4:**
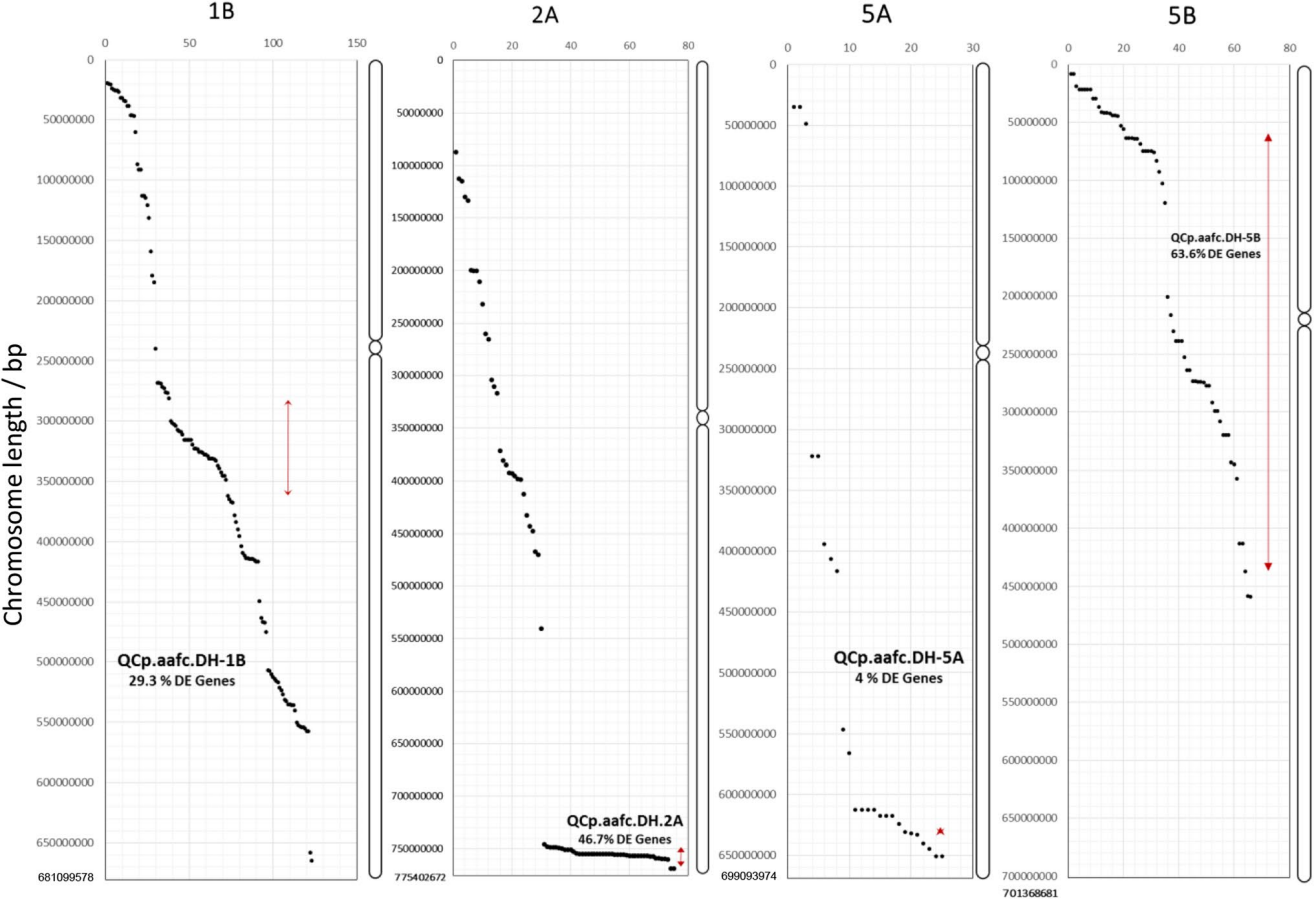
Distribution of differentially expressed genes (DEGs) identified in the pairwise comparison of DH lines grouped by the presence/absence of ergot-resistant QTL. The location of DEGs assigned to chromosomes 1B, 2A, 5A and 5B is based on the annotated gene location on the durum wheat Svevo genome reference sequence. The LOD1 interval of the ergot resistance QTL identified on each chromosome is shown by the red line. The beginning and end of chromosomes and the centromere locations are inferred from Maccaferri et al. (2019)

We cross-referenced the DEGs to the durum wheat Svevo reference genome to identify the chromosomal location of each DEG. Both LC and HC gene models were considered in this analysis. While 7.1% of the DEGs mapped to chromosome 5A, there was a much higher percentage of DEGs mapping to the three chromosomes where ergot resistance QTL were located; 1B (35.0%), 2A (21.4%) and 5B (18.8%) (Supplementary file S11). Furthermore, 36 of the DEGs located on chromosome 1B mapped within the 1B QTL interval, corresponding to 29% of the DEGs that mapped to 1B. Likewise, 35 (46.7%) DEGs on chromosome 2A and 42 (63.6%) of the DEGs on chromosome 5B mapped within the corresponding QTL interval, while only one (4.0%) DEG on chromosome 5A mapped to the QTL interval (Fig. 4; Supplementary file S11).

**Fig. 5:**
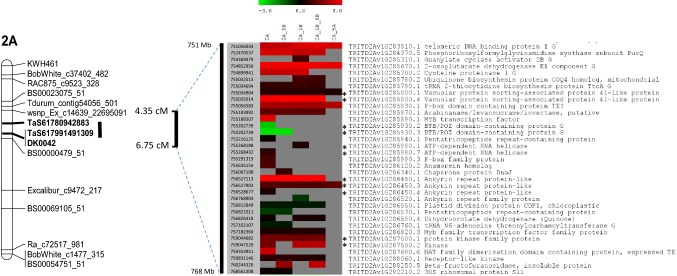
Heat map of the DEGs locating to the *QCp.aafc.DH*-*2A* QTL interval on chromosome 2A. Heat map showing Log2-fold changes of 35 DEGs that mapped to the *QCp.aafc.DH*-*2A* QTL interval. Genes down-regulated in the group of DH lines carrying *QCp.aafc.DH*-*2A* are shown in green, and genes up-regulated are shown in red. Grey boxes indicate that the gene was not significantly differentially regulated in the pairwise analysis. Reads that mapped to more than one isoform of a gene are marked with an asterisk. Svevo gene ID and annotation are shown

The 2A QTL interval was especially rich in DEGs, with 35 DEGs (Fig. 5) mapping to this 17.5-Mb region of the Svevo reference genome (Fig. 4), which contained 277 HC and 594 LC predicted genes. These 35 DEGs represent 12.6% of the HC genes and 4% of all predicted genes within the confidence interval. The vast majority of the DEGs within the *QCp.aafc.DH*-*2A* interval were up-regulated in the resistant DH lines (29 out of the 35 DEGs) and of these, 26 were more than twofold up-regulated (Supplementary file S10). A Blast2Go analysis of functional groups did not identify any significant over-representation of functional groups among these 35 DEGs.

A number of the DEGs that lie within the peak markers defining the *QCp.aafc.DH*-*2A* interval are of particular interest, including MYB transcription factors, F-box and ankyrin repeat-containing proteins, BTB/POZ-containing proteins and a variety of protein kinases. There are two HC genes annotated as MYB transcription factors (TFs) that lie near to the QTL peak markers (Fig. 5), one of which was significantly up-regulated in the GR1 v GS1 pairwise comparison. MYB TFs are a family of genes that contain a conserved MYB DNA-binding domain and are found across eukaryotes and were named originally after myeloblastosis proto-oncogenes from animals (Ambawat et al. 2013). In plants, they have been implicated in a diverse range of developmental processes, including abiotic and biotic interactions.

There were 17 F-box-containing DEGs, including two that fall within the 2A QTL interval and one within the 5B QTL interval. F-box domains were originally identified as part of the SCF ubiquitination complex (Stefanowicz et al. 2015) and are very common domains in plant proteins, having wide and varying functions (Maldonado-Calderón et al. 2012). Two ankyrin repeat-containing genes within the 2A QTL interval were differentially expressed, one of which has three isoforms. Ankyrin repeat domains are protein sequences that mediate protein–protein interactions (Bork 1993) and have been implicated in disease resistance in a number of plant species. The Arabidopsis *NPR1* gene (Non-Expressor of PR-1) contains ankyrin repeats and a BTB/POZ domain, and ankyrin repeats have been found in positive regulators of basal resistance in rice (Zhang et al. 2010, Mou et al. 2013).

Many BTB/POZ-containing proteins act as regulators of transcription, and BTB/POZ domains from several zinc finger proteins have been shown to mediate transcriptional repression (Deweindt et al. 1995). Two genes in the 2A QTL interval code for proteins with a BTB/POZ domain and interestingly, these genes are highly down-regulated in the resistant group (Supplementary file S10). Kinases catalyse the phosphorylation of specific amino acids, with numerous kinases containing proteins being implemented in plant disease resistance and signalling (Lehti-Shiu and Shiu 2012). Fourteen kinases were differentially expressed in this study, including two, up-regulated genes found within the 2A QTL interval, and three, up-regulated within the 5B QTL interval.

## Discussion

Ergot, caused by the fungal pathogen *C. purpurea,* has re-emerged in recent years as a major problem for cereal production systems, firstly because of the problems this disease causes for F_1_ hybrid seed production (Mantle and Swan 1995; Miedaner et al. 2010) and secondly because of new evidence that ergot alkaloids can find their way onto otherwise healthy grain (Gordon et al. 2019). It is anticipated that the European Union will introduce maximum limits for ergot alkaloids in processed cereal products, including wheat flour as early as 2020. This will have implications for the global cereal industry, affecting all countries trading these commodities with Europe.

Solutions to reduce ergot infection in hybrid cereal production have so far come from the deployment of pollen fertility restorer genes to maximise pollen production (with the hope that it then outcompetes infection) (Hackauf et al., 2012).Very few sources of ergot resistance per se have been identified in wheat and those that have are generally partial in effect. Four ergot resistance QTL identified in the cv. Greenshank represent a very valuable resource for wheat breeding. The QTL on 2A, *QCp.aafc.DH*-*2A*, is of particular interest because of the major effect it has on reducing honeydew production. *QCp.aafc.DH*-*1B* and *QCp.aafc.DH*-*5B* also reduced honeydew production levels, while all three QTL contributed to a reduction in sclerotia biomass. This would suggest that these QTL all act through a mechanism that directly impacts on *Cp’s* ability to grow and complete its lifecycle within the wheat flower. However, *QCp.aafc.DH*-*1B* did not significantly affect percentage infection levels, so presumably operates after infection has been achieved. *QCp.aafc.DH*-*5A* did not reduce honeydew levels or significantly affect sclerotia size, but did reduce the number of successful infections.

Differences were seen between ergot screens undertaken in Canada, where a mixture of six *Cp* isolates were used, and in the UK, where a single *Cp* isolate was used. *QCp.aafc.DH*-*5A* was detected only in the UK ergot resistance screen, so maybe an isolate-specific resistance operates that is only effective against isolate EI4. While the six isolate Canadian screen included isolate EI4, its presence in a mixture with five other isolates may not have been sufficient to see the resistance effect of *QCp.aafc.DH*-*5A*. *QCp.aafc.DH*-*1B* on the other hand was only detected in the ergot resistance screen undertaken in Canada. *QCp.aafc.DH*-*1B* may therefore not be effective against *Cp* isolate EI4, conferring resistance only to other isolate/s in the mixture. Consequently, *QCp.aafc.DH*-*5A* and *QCp.aafc.DH*-*1B* may be of less value to ergot resistance wheat breeding. This differential effect is consistent with the findings of Menzies et al. (2017) where variability in virulence phenotypes was detected in *Cp* isolates from Canada and the UK and isolates derived from other cereal and grass hosts. In that study, variation was found between 41 *Cp* isolates screened across three tetraploid and five hexaploid wheats, which resulted in 20 virulence profiles in honeydew production levels and 23 virulence profiles in total sclerotia weight.

While the ergot resistance in cv. Greenshank did not completely prevent *Cp* from completing its lifecycle, the resistance would have a significant effect on the epidemiology of the disease. *QCp.aafc.DH-2A* reduced the production of conidiospore-containing honeydew, thereby reducing inoculum and secondary spread of the disease, while all four QTL reduced sclerotia biomass (the over wintering fungal structures). Sclerotia overwinter in the soil, producing ascospores in the spring. The number of ascospores produced is proportional to the number of fruiting bodies or apothecia that germinate on the surface of the sclerotia, which is directly affected by sclerotia size (Cooke and Mitchell, 1966). Therefore, a significant reduction in sclerotia biomass would be predicted to reduce the quantity of wind-borne ascospores produced in the following spring. In addition, the QTL on 2A, 5A and 5B all reduced the percentage of successful infection. Consequently, the resistance in Greenshank would greatly impede the epidemiology of ergot, reducing the incidence of *Cp* in the following growing season.

The recent release of the tetraploid, cv. Svevo and hexaploid, cv. Chinese Spring wheat annotated genome reference sequences now enables us to investigate candidate genes underlying QTL. The MYB TFs annotated within the 2A QTL interval are of particular interest. Among the many developmental processes in which MYB TFs have been shown to be involved, hormonal regulation is of particular interest (Kranz et al. 1998). Unpublished work in the groups of Boyd and Gordon has shown *Cp* to induce significant changes in the expression of wheat genes involved in hormonal pathways, in particular gibberellic acid (GA), ethylene, auxin and jasmonic acid. In addition, two QTL for ergot resistance, (mapped in the hexaploid Robigus × Solstice double haploid population), co-located with the semi-dwarfing alleles at the *Rht* loci *Rht*-*B1* and *Rht*-*D1*, implicating a role of DELLA proteins in *Cp* infection (Gordon et al. 2015). The DELLA proteins are regulated by GA, being degraded via the 26S proteasome SCF complex in the presence of GA (Dill et al. 2004). Induction of a MYB TF on chromosome 2A may lead to altered GA levels, which in turn would alter the physiology of the plant in favour of *Cp* infection. An altered, non-functional MYB TF allele in cv. Greenshank may consequently be responsible for the ergot resistance conferred by *QCp.aafc.DH*-*2A.*

The identification of new ergot resistance in the durum wheat cv. Greenshank is very timely, given the changing political and policy landscapes around ergot sclerotia and alkaloid contamination. The health issues associated with ergot poisoning mean that grain contaminated with ergot scelrotia is discounted. Consequently, wheat breeders are interested in developing cultivars with resistance to ergot to improve farmer economic returns. As large-scale screening within a breeding program is an expensive and time-consuming exercise, marker-assisted selection of well-defined sources of ergot resistance is an appealing alternative. The resistance and associated markers identified in the current study will support the improvement in germplasm towards ergot-resistant cultivars. However, care will be needed in the deployment of these resistance loci. For example, genes that reduce ergot size could be counter-productive, with the production of smaller sclerotia being more difficult to clean from the grain. The four ergot resistance QTL identified appear to target different components of the *Cp* infection process, allowing for the deployment of resistance genes that potentially confer different modes of resistance. In addition, the genomic tools now available for tetraploid and hexaploid wheat will allow us to identify and isolate the genes responsible for these resistance QTL.

## Electronic supplementary material

Below is the link to the electronic supplementary material.

Supplementary material 1 (PDF 326 kb)

Supplementary material 2 (DOCX 20 kb)

Supplementary material 3 (DOCX 55 kb)

Supplementary material 4 (PDF 193 kb)

Supplementary material 5 (DOCX 12 kb)

Supplementary material 6 (PDF 535 kb)

Supplementary material 7 (DOCX 13 kb)

Supplementary material 8 (DOCX 13 kb)

Supplementary material 9 (PDF 205 kb)

**Supplementary file S10. Full DEG list by chromosome** Wheat differentially expressed genes associated with each ergot resistance QTL. (XLSX 175 kb)

Supplementary material 11 (DOCX 12 kb)

## Data Availability

The data that support the findings of this study are openly available in ArrayExpress: https://www.ebi.ac.uk/arrayexpress/experiments/E-MTAB-8469/, ArrayExpress accession E-MTAB-8469.
