# Supplementary material for: Genetic and transcriptional dissection of resistance to Claviceps purpurea in the durum wheat cultivar Greenshank
Source: Theor Appl Genet. 2020 Feb 14;133(6):1873–86. doi: 10.1007/s00122-020-03561-9 (PMC7237535; doi:10.1007/s00122-020-03561-9)
Supplement: Supplementary file 1 — Supplementary material 1 (PDF 326 kb) [file 122_2020_3561_MOESM1_ESM.pdf]

a

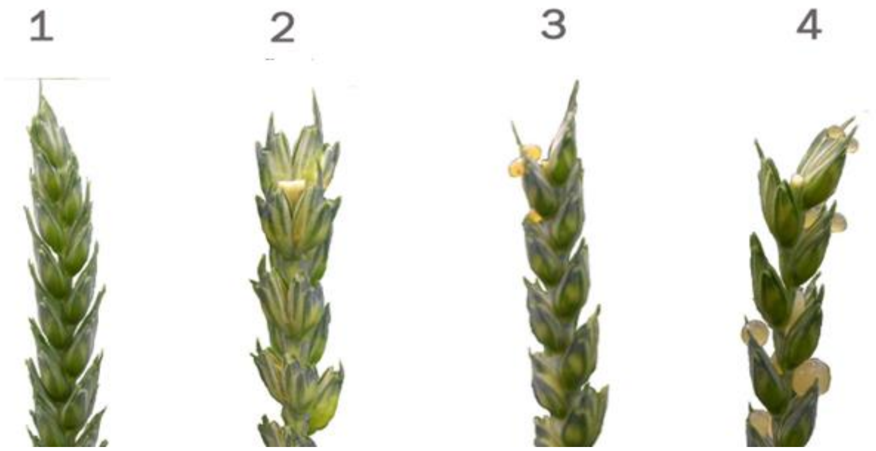


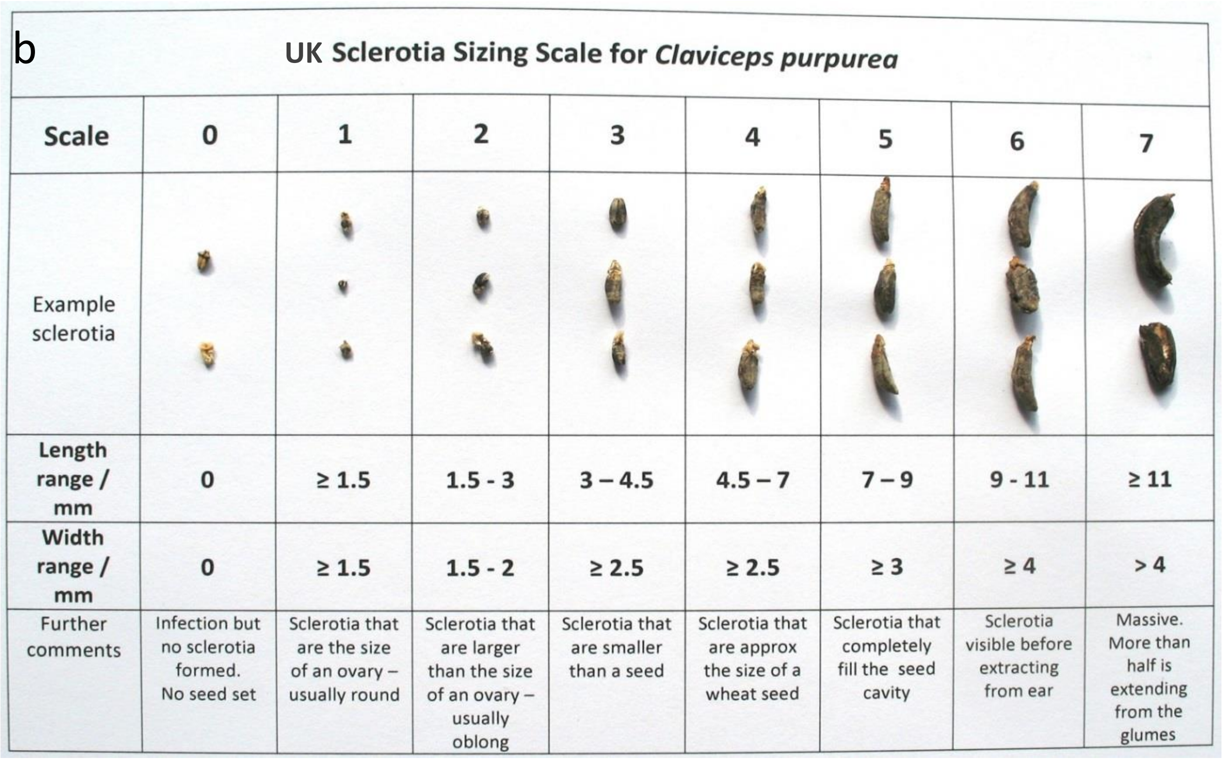

b

**UK Sclerotia Sizing Scale for *Claviceps purpurea***

| Scale | 0 | 1 | 2 | 3 | 4 | 5 | 6 | 7 |
|---|---|---|---|---|---|---|---|---|
| Example sclerotia | | | | | | | | |
| Length range / mm | 0 | ≥ 1.5 | 1.5 - 3 | 3 – 4.5 | 4.5 – 7 | 7 – 9 | 9 - 11 | ≥ 11 |
| Width range / mm | 0 | ≥ 1.5 | 1.5 - 2 | ≥ 2.5 | ≥ 2.5 | ≥ 3 | ≥ 4 | > 4 |
| Further comments | Infection but no sclerotia formed. No seed set | Sclerotia that are the size of an ovary – usually round | Sclerotia that are larger than the size of an ovary – usually oblong | Sclerotia that are smaller than a seed | Sclerotia that are approx the size of a wheat seed | Sclerotia that completely fill the seed cavity | Sclerotia visible before extracting from ear | Massive. More than half is extending from the glumes |

| UK Sclerotia sizing scale | 1 | 2 | 3 | 4 | 5 | 6 | 7 |
|---|---|---|---|---|---|---|---|
| Canadian sizing scale | | 1 | | 2 | | 3 | |

**Supplementary file S1. Ergot phenotyping scales used in the Canadian and UK ergot resistance screens.**
(a) Honeydew production scores: Each inoculated spike was scored on a scale from 1 to 4 where 1 = no honeydew, 2 = honeydew confined to glumes, 3 = honeydew exuding from the flowers in small droplets, and 4 = large droplets of honeydew and/or honeydew running down the spike. (b) Ergot sclerotia sizing scales used in the UK and Canada. 0 indicated an infection where no sclerotia formed, leaving only a dried out ovary.
