# Supplementary material for: Genetic and transcriptional dissection of resistance to Claviceps purpurea in the durum wheat cultivar Greenshank
Source: Theor Appl Genet. 2020 Feb 14;133(6):1873–86. doi: 10.1007/s00122-020-03561-9 (PMC7237535; doi:10.1007/s00122-020-03561-9)
Supplement: Supplementary file 2 — Supplementary material 2 (DOCX 20 kb) [file 122_2020_3561_MOESM2_ESM.docx]

| **ID** | **Chr** | **Allele X Primer** | **Allele Y Primer** | **Common Primer** | **X** | **Y** |
| --- | --- | --- | --- | --- | --- | --- |
| KWH 461 | 2A | GAAGGTGACCAAGTTCATGCTAACCTTACACCTGAATGTAAGAAGTTC | GAAGGTCGGAGTCAACGGATTAACCTTACACCTGAATGTAAGAAGTTG | GGATAGGACATGTCCCCAAGAAACAT | C | G |
| KWH 463 | 2A | GAAGGTGACCAAGTTCATGCTCCTGTTTGCTTGTCTCTCTAACATGT | GAAGGTCGGAGTCAACGGATTCTGTTTGCTTGTCTCTCTAACATGC | ATCTCTTCATCAGAAGGCCAAAGCATTT | T | C |
| DK0017 | 2A | agctctgctgcgataagtttaaatC | agctctgctgcgataagtttaaatT | tgcaccatctgttacattgcc | C | T |
| DK0025 | 2A | gcaaagagtcataatccccgaatC | gcaaagagtcataatccccgaatT | acgtctgaggccacataaca | C | T |
| DK0029 | 2A | ttgcacgccttctttgtagC | ttgcacgccttctttgtagT | tgctgttgcttcatcagtagga | C | T |
| DK0036 | 2A | acattacagatggtgttgtggA | acattacagatggtgttgtggG | acgacgaaacacgacacgat | A | G |
| DK0037 | 2A | gtcgtcgtggtcgtttgttT | gtcgtcgtggtcgtttgttC | cctgcatcacacagaccact | T | C |
| DK0042 | 2A | cctagcaaagcctgagcattattT | cctagcaaagcctgagcattattA | tgtattcgcctggttgaacc | T | A |
| DK0045 | 2A | gggcctgtgtgctataagagtaA | gggcctgtgtgctataagagtaG | aatccggatgtgctaggtgt | A | G |
| DK0050 | 2A | tcaaagacatgagctgctatcT | tcaaagacatgagctgctatcC | gcatgagtgggtaggaaaggt | T | C |
| DK0077 | 2A | catgaaatgcctcaagttaatttcG | catgaaatgcctcaagttaatttcC | gcaacccaatgtcgcaactt | G | C |
| TaS26027532_510 | 5B | GTTGGACTTCATCTCCCATAGCG | AGTTGGACTTCATCTCCCATAGCA | TGATCAAAGGCTGTGCTGGGAAGTT | C | T |
| TaS19222949_285 | 1B | CCACGTCCAAGAAGCAGGCC | CCACGTCCAAGAAGCAGGCG | CTCCGCAGCCGCGTGTGCAT | C | G |
| TaS22378302_168 | 1B | GTCAACGACCTCGGCGTCG | GTCAACGACCTCGGCGTCC | TCCAGCACTCCCGCTCCGTTA | C | G |
| TaS37828549_230 | 1B | CCTCGCTGCCGCGGCCT | CTCGCTGCCGCGGCCC | CCGTGGAGTCGCGGGATTTCAT | A | G |
| TaS52543436_853 | 1B | CGGACCGAGCTCACTGAGG | GTCGGACCGAGCTCACTGAGA | GGTTGGATGATGACAAGGCGGTT | C | T |
| TaS52544545_1264 | 1B | CTGTTCCAGCAAAACTGGGC | CTCTGTTCCAGCAAAACTGGGG | GGCCATCTGATTTATAGGCCCATGAT | C | G |
| TaS58864761_1142 | 1B | GAGGAGCAGGCCTCTGCG | GGAGGAGCAGGCCTCTGCA | CGGTGGGATGAAACGAAGCAAACAA | C | T |
| TaS61626548_1219 | 1B | GAAATCATTACACACTGTAGCGAGAT | GAAATCATTACACACTGTAGCGAGAG | GTACGCTGGATTTCAGTCCGTTGAA | A | C |
| TaS61667965_174 | 1B | GGCTAATCTTGAAGGAGCAGAAATAG | GGCTAATCTTGAAGGAGCAGAAATAA | ATGGACGGAGACGGAGAGATAACAT | C | T |
| TaS61763935_365 | 1B | GAAGAGCCGGCTGGCGGTT | GAAGAGCCGGCTGGCGGTA | GCCGAAGCCGCCTCCGATAAAT | A | T |
| TaS61779084_1153 | 1B | ACCGCTAATAGCGAAATTCACTCCA | CGCTAATAGCGAAATTCACTCCG | CCCTTATCTGAAGGCAATTGGTTCTAATT | A | G |
| TaS61780753_981 | 1B | TCAGTCTGAACCTCTCCGTG | CTTCAGTCTGAACCTCTCCGTC | GAGGACGAAGTATCCAGCTGGCT | C | G |
| TaS61829703_610 | 1B | CAAAGGTCTTGTTCCATGTCGCT | CAAAGGTCTTGTTCCATGTCGCC | AATATGTTGGATGCGTAGAAGATCGTCTT | A | G |
| TaS65606418_261 | 1B | AGATCATGTGGAGCTGCCTCG | GAGATCATGTGGAGCTGCCTCA | AGTTGCCTACCGGCGCGAGAAT | C | T |
| TaS61832467_2424 | 1B | ATTTATTATCGATTAATGTAACACTTTACG | CCTATTTATTATCGATTAATGTAACACTTTACT | CGGCCCATTGCCAGCATCTACAT | G | T |
| TaS17893645_202 | 5A | AAGAATCCAGACACATCTGTCTGC | GAAGAATCCAGACACATCTGTCTGT | AGCGGTCACTTAACCAAAAGCGCAA | C | T |
| TaS52543119_1835 | 5A | GGCGTGGGACACCTTCGAC | GGGCGTGGGACACCTTCGAT | GCCCGCTCCTGCTCCGAGTA | C | T |
| TaS52546643_1272 | 5A | TGCACCTTGAAGCTAGTTACCTTC | CTTGCACCTTGAAGCTAGTTACCTTT | GTCTTCCTCCATCCCTCGGCAA | C | T |
| TaS61801987_699 | 5A | AGCAACCAACTTACCAAAAGTTGATTG | CAGCAACCAACTTACCAAAAGTTGATTA | GACTGAGTTTCTTCAGATTAGCATGCTTT | C | T |
| TaS61811944_2760 | 5A | CACAATTTCTGCCGATAGTCTACAAG | CACAATTTCTGCCGATAGTCTACAAT | TACAGACAGCCCCAACCTTTACCTA | G | T |
| TaS61602624_507 | 2A | GAAGAAGTTTGTGGAGCTCTTTCTCA | AAGAAGTTTGTGGAGCTCTTTCTCG | TGGATCGTCGTGCTTACCTGGTTT | A | G |
| TaS52545345_2276 | 2A | GAGTATTTGGAGGAGGCATTCACA | GAGTATTTGGAGGAGGCATTCACT | CTTGTATATGCGCACTATCCAGTTTGAT | A | T |
| TaS52541421_419 | 2A | GGCGAGCAAGATGTCGCCGT | GCGAGCAAGATGTCGCCGG | GCCGTGGCCTACGAGGACAAT | A | C |
| TaS52543454_1001 | 2A | AGAAGTACTTCATCCGCCTCAAC | CAGAAGTACTTCATCCGCCTCAAT | ATCGCCGCCTCTCCCTGTTCAT | C | T |
| TaS61780942_883 | 2A | TGTAGGAGATTGTGAGGTGGGAA | GTAGGAGATTGTGAGGTGGGAG | GCAGTTCTTGCACGCCTTCTTTGTA | A | G |
| TaS61799149_1309 | 2A | CAGTACTTTTGTTATTGTCTGGTAATCAC | CAGTACTTTTGTTATTGTCTGGTAATCAT | CCGCCTTCATCTACTGTTACAGCAA | C | T |
| TaS65622867_376 | 2A | GGGGCCGGCGCGGTAAC | CGGGGCCGGCGCGGTAAA | CTTCCTGCATGCCTGCTCCGAT | G | T |

**Supplementary file S2**. Markers developed as part of this study to further populate the QTL intervals. Markers with the suffix KWH and DK were developed by Colin Hiebert and David Konkin, respectively, in Canada. SNPs for markers beginning TaS were identified by Gary Barker in the UK, and were developed by Anna Gordon and LGC Genomics. The location of these markers on the linkage maps of the DH population can be found in Supplementary file S6
