# Supplementary material for: Genetic and transcriptional dissection of resistance to Claviceps purpurea in the durum wheat cultivar Greenshank
Source: Theor Appl Genet. 2020 Feb 14;133(6):1873–86. doi: 10.1007/s00122-020-03561-9 (PMC7237535; doi:10.1007/s00122-020-03561-9)
Supplement: Supplementary file 3 — Supplementary material 3 (DOCX 55 kb) [file 122_2020_3561_MOESM3_ESM.docx]

**Supplementary file S3**. Summary of seventeen double haploid (DH) lines selected for their ergot resistance phenotypes, and resistant or susceptible alleles at each ergot resistance QTL identified in the Greenshank_RIL3 x AC Avonlea (DH) population. Rec. indicates a recombination event in the QTL interval.
