# Supplementary material for: Genetic and transcriptional dissection of resistance to Claviceps purpurea in the durum wheat cultivar Greenshank
Source: Theor Appl Genet. 2020 Feb 14;133(6):1873–86. doi: 10.1007/s00122-020-03561-9 (PMC7237535; doi:10.1007/s00122-020-03561-9)
Supplement: Supplementary file 4 — Supplementary material 4 (PDF 193 kb) [file 122_2020_3561_MOESM4_ESM.pptx]

## Slide 1
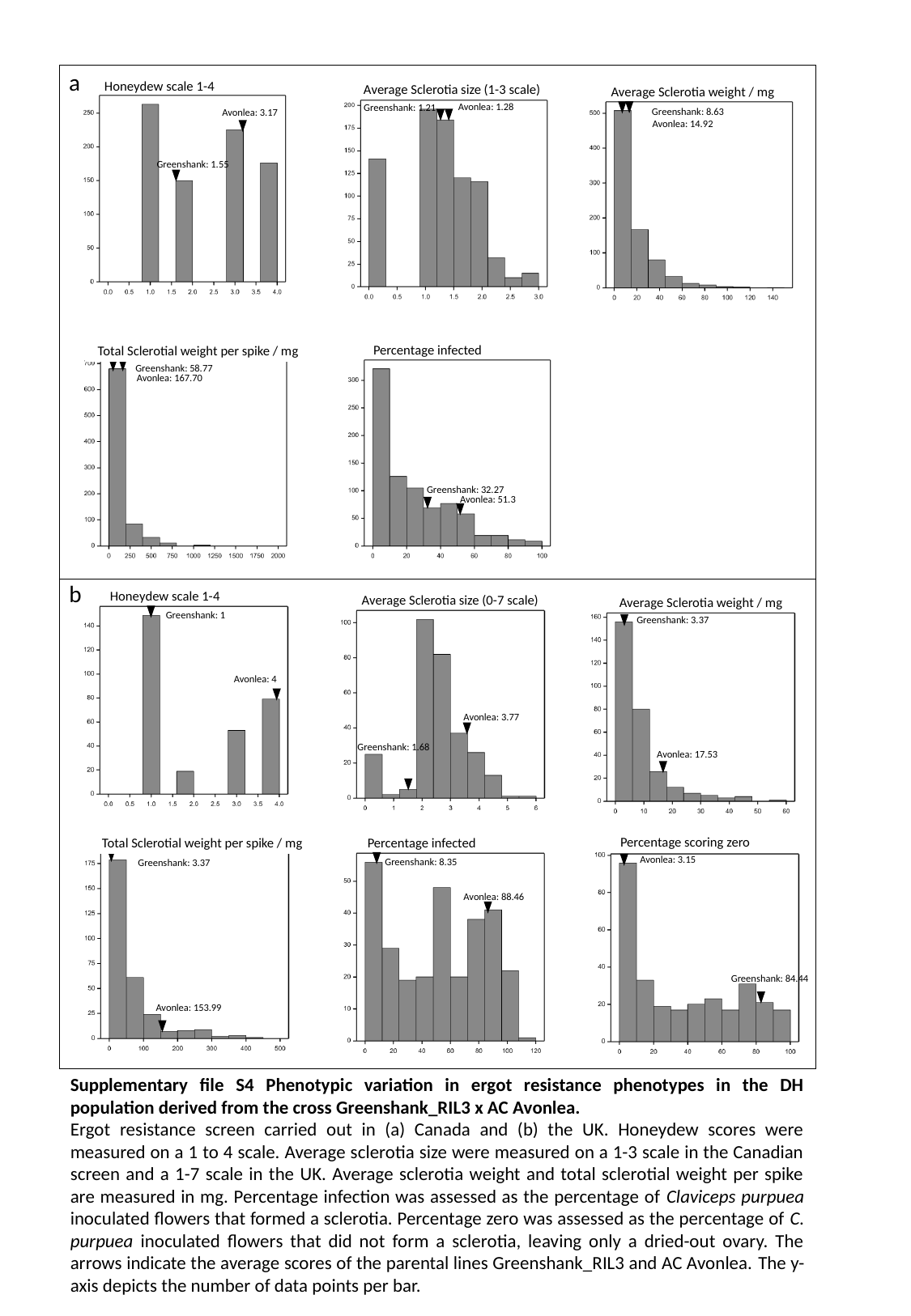

Avonlea: 3.17
Avonlea: 3.17
1960B-173A 1.55
Greenshank: 1.55
Avonlea: 1.28
Avonlea: 1.28
Greenshank: 1.21
1960B-173A: 1.21
1960B-173A: 8.63
Avonlea: 14.92
Greenshank: 8.63
Avonlea: 14.92
a
1960B-173A: 58.77
Avonlea: 167.70
Greenshank: 58.77
Avonlea: 167.70
1960B-173A: 32.27
Avonlea: 51.3
Greenshank: 32.27
Avonlea: 51.3
Avonlea: 3.77
Avonlea: 3.77
1960B-173A: 1.68
Greenshank: 1.68
1960B-173A: 1
Greenshank: 1
Avonlea: 4
Avonlea: 4
b
1960B-173A: 3.37
Greenshank: 3.37
Avonlea: 17.53
Avonlea: 17.53
1960B-173A: 3.37
Greenshank: 3.37
Avonlea: 153.99
Avonlea: 153.99
Greenshank: 8.35
Avonlea: 88.46
Avonlea: 88.46
Avonlea: 88.46
Avonlea: 3.15
Greenshank: 84.44
1960B-173A: 84.44
Supplementary file S4 Phenotypic variation in ergot resistance phenotypes in the DH population derived from the cross Greenshank_RIL3 x AC Avonlea.
Ergot resistance screen carried out in (a) Canada and (b) the UK. Honeydew scores were measured on a 1 to 4 scale. Average sclerotia size were measured on a 1-3 scale in the Canadian screen and a 1-7 scale in the UK. Average sclerotia weight and total sclerotial weight per spike are measured in mg. Percentage infection was assessed as the percentage of Claviceps purpuea inoculated flowers that formed a sclerotia. Percentage zero was assessed as the percentage of C. purpuea inoculated flowers that did not form a sclerotia, leaving only a dried-out ovary. The arrows indicate the average scores of the parental lines Greenshank_RIL3 and AC Avonlea. The y-axis depicts the number of data points per bar.
Honeydew scale 1-4
Average Sclerotia size (1-3 scale)
Average Sclerotia weight / mg
Total Sclerotial weight per spike / mg
Percentage infected
Honeydew scale 1-4
Average Sclerotia size (0-7 scale)
Average Sclerotia weight / mg
Total Sclerotial weight per spike / mg
Percentage scoring zero
Percentage infected
