# Supplementary material for: Genetic and transcriptional dissection of resistance to Claviceps purpurea in the durum wheat cultivar Greenshank
Source: Theor Appl Genet. 2020 Feb 14;133(6):1873–86. doi: 10.1007/s00122-020-03561-9 (PMC7237535; doi:10.1007/s00122-020-03561-9)
Supplement: Supplementary file 5 — Supplementary material 5 (DOCX 12 kb) [file 122_2020_3561_MOESM5_ESM.docx]

| **Phenotypic data set** | **Phenotypic trait** | **H^2^** |
| --- | --- | --- |
| **HD_RIL_Can** | **HD** | **0.95** |
| **HD_DH_Can** | **HD** | **0.76** |
| **HD_DH_UK** | **HD** | **0.92** |
| **TW_RIL_Can** | **TW** | **0.87** |
| **TW_DH_Can** | **TW** | **0.13** |
| **TW_DH_UK** | **TW** | **0.79** |
| **SW_RIL_Can** | **SW** | **0.77** |
| **SW_DH_Can** | **SW** | **0.13** |
| **SW_DH_UK** | **SW** | **0.64** |
| **SS_RIL_Can** | **SS** | **0.81** |
| **SS_DH_Can** | **SS** | **0.15** |
| **SS_DH_UK** | **SS** | **0.73** |
| **%Inf_RIL_Can** | **%Inf** | **0.92** |
| **%Inf_DH_Can** | **%Inf** | **0.13** |
| **%Inf_DH_UK** | **%Inf** | **0.84** |
| **%Zero_DH_UK** | **%Zero** | **0.90** |

**Supplementary file S5.** Broad sense heritability values (H^2^) for honeydew production (HD), total sclerotia weight (TW), average sclerotia weight (SW), and average sclerotia size (SS) per spike, percentage infection (%Inf) and percentage zero (%zero). Screens were undertaken in Canada (Can) or in the UK (UK) on the Recombinant Inbred Line or Doubled Haploid populations.
