# Supplementary material for: Genetic and transcriptional dissection of resistance to Claviceps purpurea in the durum wheat cultivar Greenshank
Source: Theor Appl Genet. 2020 Feb 14;133(6):1873–86. doi: 10.1007/s00122-020-03561-9 (PMC7237535; doi:10.1007/s00122-020-03561-9)
Supplement: Supplementary file 6 — Supplementary material 6 (PDF 535 kb) [file 122_2020_3561_MOESM6_ESM.pdf]

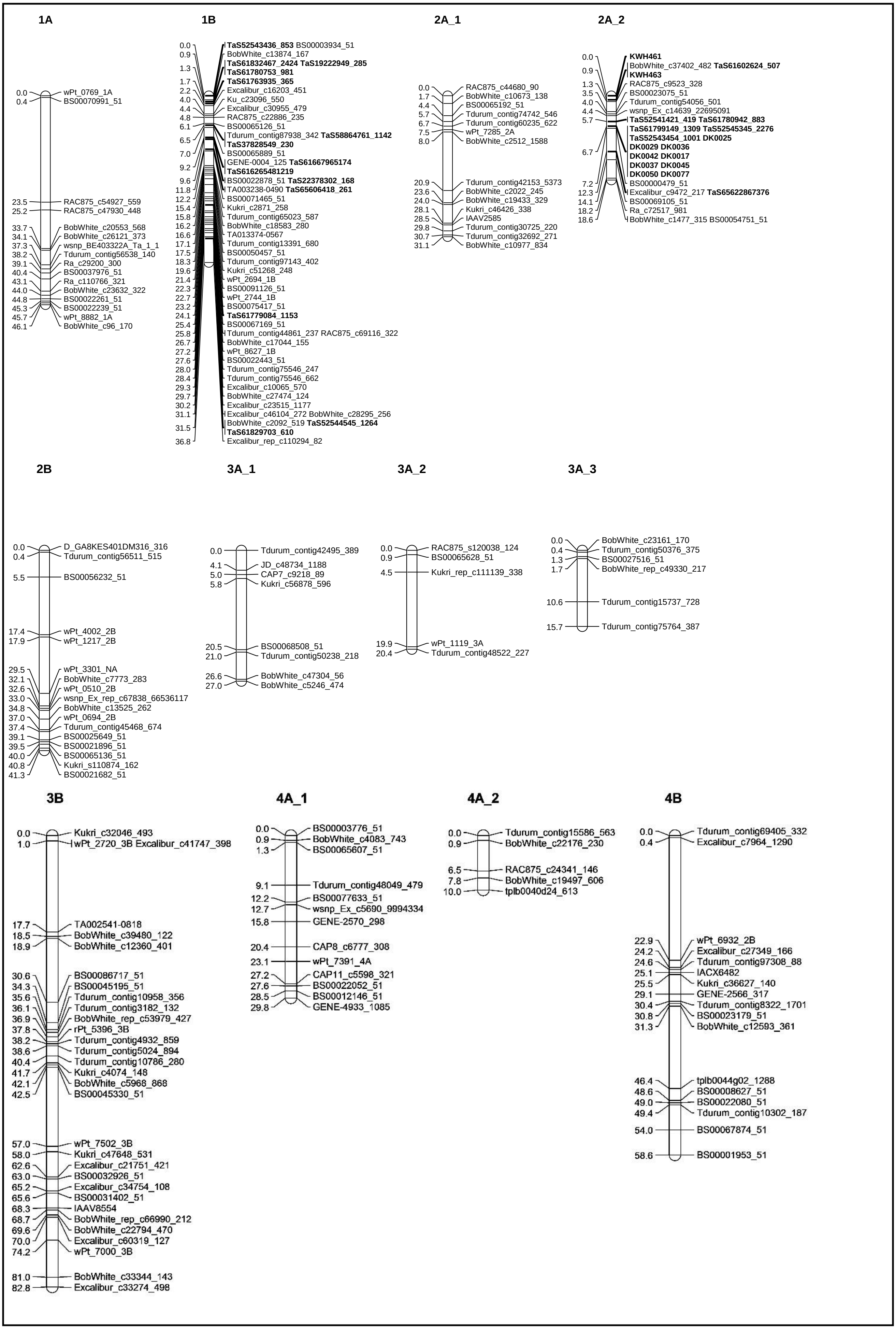
1A
0.0 wPt_0769_1A
0.4 BS00070991_51
23.5 RAC875_c54927_559
25.2 RAC875_c47930_448
33.7 BobWhite_c20553_568
34.1 BobWhite_c26121_373
37.3 wsnp_BE403322A_Ta_1_1
38.2 Tdurum_contig56538_140
39.1 Ra_c29200_300
40.4 BS00037976_51
43.1 Ra_c110766_321
44.0 BobWhite_c23632_322
44.8 BS00022261_51
45.3 BS00022239_51
45.7 wPt_8882_1A
46.1 BobWhite_c96_170
1B
0.0 TaS52543436_853 BS00003934_51
0.9 BobWhite_c13874_167
1.3 TaS61832467_2424 TaS19222949_285
1.7 TaS61780753_981
2.2 TaS61763935_365
4.0 Excalibur_c16203_451
4.4 Ku_c23096_550
4.8 Excalibur_c30955_479
6.1 RAC875_c22886_235
6.5 BS00065126_51
7.0 Tdurum_contig87938_342 TaS58864761_1142
9.2 TaS37828549_230
9.6 BS00065889_51
11.8 GENE-0004_125 TaS61667965174
12.2 TaS616265481219
15.4 BS00022878_51 TaS22378302_168
15.8 TA003238-0490 TaS65606418_261
16.2 BS00071465_51
16.6 Kukri_c2871_258
17.1 Tdurum_contig65023_587
17.5 BobWhite_c18583_280
18.3 TA013374-0567
19.6 Tdurum_contig13391_680
21.4 BS00050457_51
22.3 Tdurum_contig97143_402
22.7 Kukri_c51268_248
23.2 wPt_2694_1B
24.1 BS00091126_51
25.4 wPt_2744_1B
25.8 BS00075417_51
26.7 TaS61779084_1153
27.2 BS00067169_51
27.6 Tdurum_contig44861_237 RAC875_c69116_322
28.0 BobWhite_c17044_155
28.4 wPt_8627_1B
29.3 BS00022443_51
29.7 Tdurum_contig75546_247
30.2 Tdurum_contig75546_662
31.1 Excalibur_c10065_570
31.5 BobWhite_c27474_124
36.8 Excalibur_c23515_1177
Excalibur_c46104_272 BobWhite_c28295_256
BobWhite_c2092_519 TaS52544545_1264
TaS61829703_610
Excalibur_rep_c110294_82
2A_1
0.0 RAC875_c44680_90
1.7 BobWhite_c10673_138
4.4 BS00065192_51
5.7 Tdurum_contig74742_546
6.7 Tdurum_contig60235_622
7.5 wPt_7285_2A
8.0 BobWhite_c2512_1588
20.9 Tdurum_contig42153_5373
23.6 BobWhite_c2022_245
24.0 BobWhite_c19433_329
28.1 Kukri_c46426_338
28.5 IAAV2585
29.8 Tdurum_contig30725_220
30.7 Tdurum_contig32692_271
31.1 BobWhite_c10977_834
2A_2
0.0 KWH461
0.9 BobWhite_c37402_482 TaS61602624_507
1.3 KWH463
3.5 RAC875_c9523_328
4.0 BS00023075_51
4.4 Tdurum_contig54056_501
5.7 wsnp_Ex_c14639_22695091
6.7 TaS52541421_419 TaS61780942_883
7.2 TaS61799149_1309 TaS52545345_2276
12.3 TaS52543454_1001 DK0025
14.1 DK0029 DK0036
18.2 DK0042 DK0017
18.6 DK0037 DK0045
DK0050 DK0077
BS00000479_51
Excalibur_c9472_217 TaS65622867376
BS00069105_51
Ra_c72517_981
BobWhite_c1477_315 BS00054751_51
2B
0.0 D_GA8KES401DM316_316
0.4 Tdurum_contig56511_515
5.5 BS00056232_51
17.4 wPt_4002_2B
17.9 wPt_1217_2B
29.5 wPt_3301_NA
32.1 BobWhite_c7773_283
32.6 wPt_0510_2B
33.0 wsnp_Ex_rep_c67838_66536117
34.8 BobWhite_c13525_262
37.0 wPt_0694_2B
37.4 Tdurum_contig45468_674
39.1 BS00025649_51
39.5 BS00021896_51
40.0 BS00065136_51
40.8 Kukri_s110874_162
41.3 BS00021682_51
3A_1
0.0 Tdurum_contig42495_389
4.1 JD_c48734_1188
5.0 CAP7_c9218_89
5.8 Kukri_c56878_596
20.5 BS00068508_51
21.0 Tdurum_contig50238_218
26.6 BobWhite_c47304_56
27.0 BobWhite_c5246_474
3A_2
0.0 RAC875_s120038_124
0.9 BS00065628_51
4.5 Kukri_rep_c111139_338
19.9 wPt_1119_3A
20.4 Tdurum_contig48522_227
3A_3
0.0 BobWhite_c23161_170
0.4 Tdurum_contig50376_375
1.3 BS00027516_51
1.7 BobWhite_rep_c49330_217
10.6 Tdurum_contig15737_728
15.7 Tdurum_contig75764_387
3B
0.0 Kukri_c32046_493
1.0 wPt_2720_3B Excalibur_c41747_398
17.7 TA002541-0818
18.5 BobWhite_c39480_122
18.9 BobWhite_c12360_401
30.6 BS00086717_51
34.3 BS00045195_51
35.6 Tdurum_contig10958_356
36.1 Tdurum_contig3182_132
36.9 BobWhite_rep_c53979_427
37.8 rPt_5396_3B
38.2 Tdurum_contig4932_859
38.6 Tdurum_contig5024_894
40.4 Tdurum_contig10786_280
41.7 Kukri_c4074_148
42.1 BobWhite_c5968_868
42.5 BS00045330_51
57.0 wPt_7502_3B
58.0 Kukri_c47648_531
62.6 Excalibur_c21751_421
63.0 BS00032926_51
65.2 Excalibur_c34754_108
65.6 BS00031402_51
68.3 IAAV8554
68.7 BobWhite_rep_c66990_212
69.6 BobWhite_c22794_470
70.0 Excalibur_c60319_127
74.2 wPt_7000_3B
81.0 BobWhite_c33344_143
82.8 Excalibur_c33274_498
4A_1
0.0 BS00003776_51
0.9 BobWhite_c4083_743
1.3 BS00065607_51
9.1 Tdurum_contig48049_479
12.2 BS00077633_51
12.7 wsnp_Ex_c5690_9994334
15.8 GENE-2570_298
20.4 CAP8_c6777_308
23.1 wPt_7391_4A
27.2 CAP11_c5598_321
27.6 BS00022052_51
28.5 BS00012146_51
29.8 GENE-4933_1085
4A_2
0.0 Tdurum_contig15586_563
0.9 BobWhite_c22176_230
6.5 RAC875_c24341_146
7.8 BobWhite_c19497_606
10.0 tplb0040d24_613
4B
0.0 Tdurum_contig69405_332
0.4 Excalibur_c7964_1290
22.9 wPt_6932_2B
24.2 Excalibur_c27349_166
24.6 Tdurum_contig97308_88
25.1 IACX6482
25.5 Kukri_c36627_140
29.1 GENE-2566_317
30.4 Tdurum_contig8322_1701
30.8 BS00023179_51
31.3 BobWhite_c12593_361
46.4 tplb0044g02_1288
48.6 BS00008627_51
49.0 BS00022080_51
49.4 Tdurum_contig10302_187
54.0 BS00067874_51
58.6 BS00001953_51

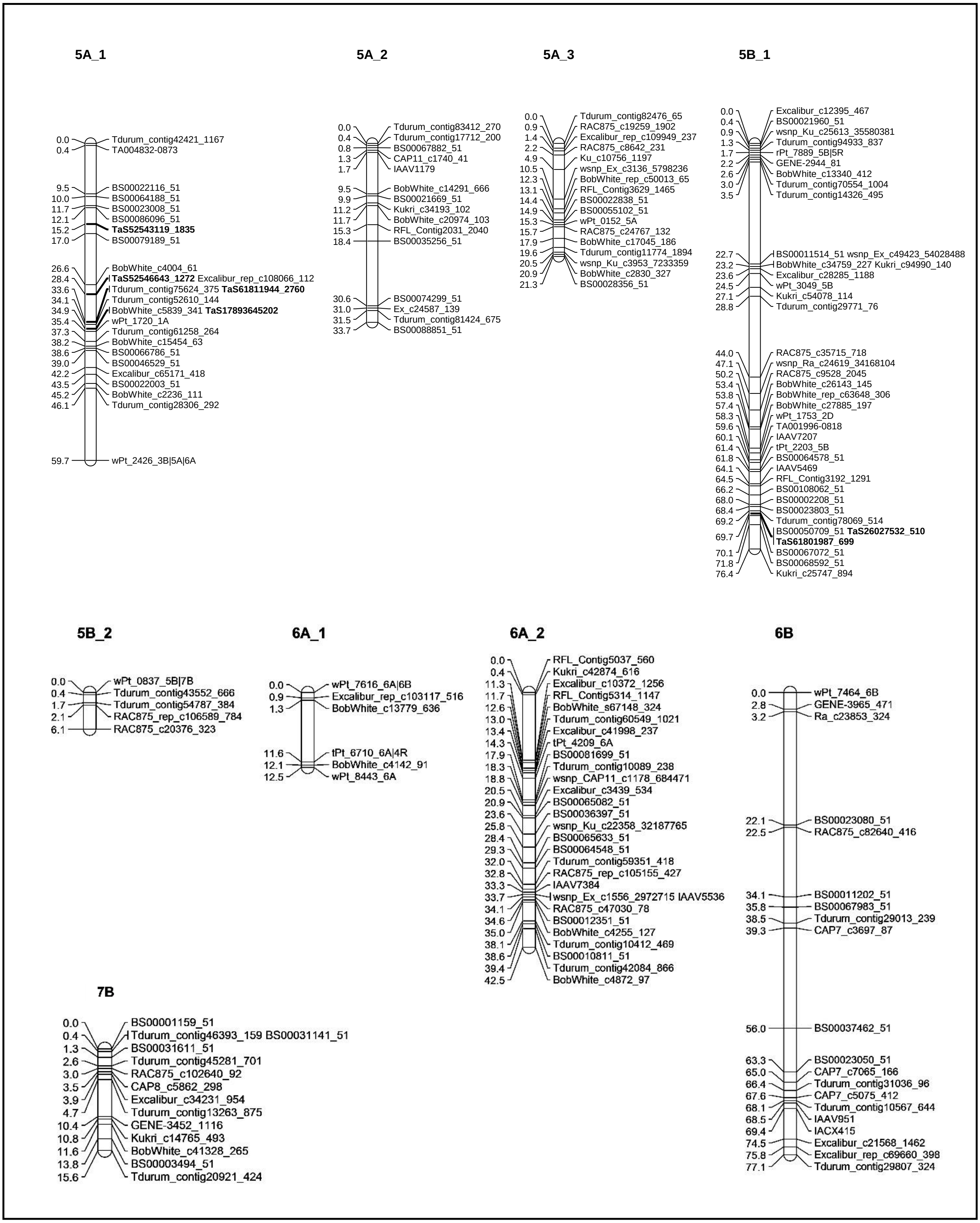

Supplementary file S6. The Greenshank_RIL3 x AC Avonlea DH population linkage map.

The map consists of 357 iSelect 90k markers and KASP markers developed in this study. The KASPs are denoted in bold typeface with the suffix TaS, DK or KMH.
