# Supplementary material for: Genetic and transcriptional dissection of resistance to Claviceps purpurea in the durum wheat cultivar Greenshank
Source: Theor Appl Genet. 2020 Feb 14;133(6):1873–86. doi: 10.1007/s00122-020-03561-9 (PMC7237535; doi:10.1007/s00122-020-03561-9)
Supplement: Supplementary file 7 — Supplementary material 7 (DOCX 13 kb) [file 122_2020_3561_MOESM7_ESM.docx]

| Resistant group name | Resistant QTL present | DH lines | Susceptible group name | Resistant QTL absent | DH lines | Pairwise comparisons |  |
| --- | --- | --- | --- | --- | --- | --- | --- |
| GR1 | 2A | DH_23  DH_28  DH_30  DH_35  DH_41  DH_52  DH_69  DH_82  DH_88 | GS1 | null for 2A | DH_10  DH_21  DH_27  DH_56  DH_59  DH_64  DH_73  DH_116 | GR1 v GS1 | To identify DEGs associated with the QTL on chromosome 2A |
| GR2 | 2A, 5B | DH_23  DH_30  DH_52  DH_69  DH_82  DH_88 | GS2 | null for 2A, 5B, | DH_21  DH_56  DH_59  DH_64  DH_73  DH_116 | GR2 v GS2 | To identify DEGs associated with the QTL on chromosomes 2A and 5B |
| GR3 | 1B, 2A | DH_23  DH_28  DH_30  DH_35  DH_69  DH_82 | GS3 | null for 1B, 2A | DH_64  DH_73  DH_116 | GR3 v GS3 | To identify DEGs associated with the QTL on chromosomes 2A and 1B |
| GR4 | 2A, 5A | DH_23  DH_30  DH_35  DH_69  DH_82  DH_88 | GS4 | null for 2A, 5A | DH_10  DH_27  DH_64 | GR4 v GS4 | To identify DEGs associate with the QTL on chromosomes 2A and 5A |
| GR5 | 1B, 2A, 5B | DH_23  DH_30  DH_69  DH_82 | GS5 | null for 1B, 2A, 5B, | DH_64  DH_73  DH_116 | GR5 v GS5 | To identify DEGs associated with the QTL on chromosomes 2A, 1B and 5B |
|  | |  |  |  |  |  |  |

**Supplementary file S7.** The pairwise group comparisons used to identify differentially expressed wheat genes (DEGs). Ergot resistant and susceptible doubled haploid (DH) lines grouped according to the ergot resistance QTL present.
