# Supplementary material for: Genetic and transcriptional dissection of resistance to Claviceps purpurea in the durum wheat cultivar Greenshank
Source: Theor Appl Genet. 2020 Feb 14;133(6):1873–86. doi: 10.1007/s00122-020-03561-9 (PMC7237535; doi:10.1007/s00122-020-03561-9)
Supplement: Supplementary file 8 — Supplementary material 8 (DOCX 13 kb) [file 122_2020_3561_MOESM8_ESM.docx]

| Pairwise comparisons | QTL combination | Total DEG | Total  Number of DEGs up- and down-regulated | | Number of DEG mapping to 1B | Number of DEGs with a L2FC of </>1  1B | | Number of DEGs mapping to 2A | Number of DEGs with a L2FC of </>1  2A | | Number of DEGs mapping to 5A | Number of DEGs with a L2FC of </>1  5A | | Number of DEGs mapping to 5B | Number of DEGs with a L2FC of </>1  5B | |
| --- | --- | --- | --- | --- | --- | --- | --- | --- | --- | --- | --- | --- | --- | --- | --- | --- |
|  |  |  | Up in Sus | Up in Res |  | Up in Sus | Up in Res |  | Up in Sus | Up in Res |  | Up in Sus | Up in Res |  | Up in Sus | Up in Res |
| GR1 v GS1 | 2A v null for 2A | 70 | 26 | 35 | 1 | 1 | 0 | 49 | 14 | 27 | 3 | 1 | 2 | 6 | 0 | 6 |
| GR2 v GS2 | 2A+5B v null for 2A+5B | 133 | 60 | 62 | 2 | 1 | 1 | 40 | 13 | 22 | 7 | 1 | 5 | 62 | 33 | 29 |
| GR3 v GS3 | 2A+1B v null for 2A+1B | 202 | 96 | 96 | 114 | 58 | 54 | 51 | 14 | 31 | 0 | 0 | 0 | 2 | 2 | 0 |
| GR4 v GS4 | 2A+5A v null for 2A + 5A | 27 | 12 | 15 | 2 | 2 | 0 | 8 | 2 | 6 | 13 | 8 | 5 | 0 | 0 | 0 |
| GR5 v GS5 | 2A+1B+5B v null for 2A+1B+5B | 249 | 122 | 110 | 110 | 57 | 51 | 43 | 12 | 24 | 6 | 1 | 2 | 50 | 27 | 23 |

**Supplementary file S8.** Summary of the outputs of the RNASeq pairwise comparisons showing the numbers of differentially expressed genes (DEGs) up- and down-regulated at 48 hours after inoculation with *Claviceps purpurea,* and the numbers of DEGs mapping to the durum wheat Svevo reference chromosomes 1B, 2A, 5A and 5B.
