# Supplementary material for: Genetic and transcriptional dissection of resistance to Claviceps purpurea in the durum wheat cultivar Greenshank
Source: Theor Appl Genet. 2020 Feb 14;133(6):1873–86. doi: 10.1007/s00122-020-03561-9 (PMC7237535; doi:10.1007/s00122-020-03561-9)
Supplement: Supplementary file 11 — Supplementary material 11 (DOCX 12 kb) [file 122_2020_3561_MOESM11_ESM.docx]

|  | Number of DEGs mapping to each chromosome | % of DEGs mapping to each chromosome | Number of DEGs that mapped into the QTL 1LOD interval(s) | % of total DEGs that mapped into the QTL LOD1 interval |
| --- | --- | --- | --- | --- |
| 1A | 12 | 3.4 |  |  |
| 1B | 123 | 35.0 | 36 (29.3%) | 10.3 |
| 2A | 75 | 21.4 | 35 (46.7%) | 10.0 |
| 2B | 10 | 2.9 |  |  |
| 3A | 2 | 0.6 |  |  |
| 3B | 6 | 1.7 |  |  |
| 4A | 5 | 1.4 |  |  |
| 4B | 3 | 0.9 |  |  |
| 5A | 25 | 7.1 | 1 (4%) | 0.3 |
| 5B | 66 | 18.8 | 42 (63.6%) | 11.9 |
| 6A | 2 | 0.6 |  |  |
| 6B | 4 | 1.1 |  |  |
| 7A | 4 | 1.1 |  |  |
| 7B | 4 | 1.1 |  |  |
| ChrUn | 10 | 2.9 |  |  |
| Total | 351 | 100 | 114 | 32.5 |

**Supplementary file S11.** Numbers of differentially expressed genes (DEGs) that mapped to the durum wheat Svevo genome reference chromosomes, and within the ergot resistance QTL intervals.
