# Supplementary material for: Genetic and transcriptional dissection of resistance to Claviceps purpurea in the durum wheat cultivar Greenshank
Source: Theor Appl Genet. 2020 Feb 14;133(6):1873–86. doi: 10.1007/s00122-020-03561-9 (PMC7237535; doi:10.1007/s00122-020-03561-9)
Supplement: Supplementary file 9 — Supplementary material 9 (PDF 205 kb) [file 122_2020_3561_MOESM9_ESM.pdf]

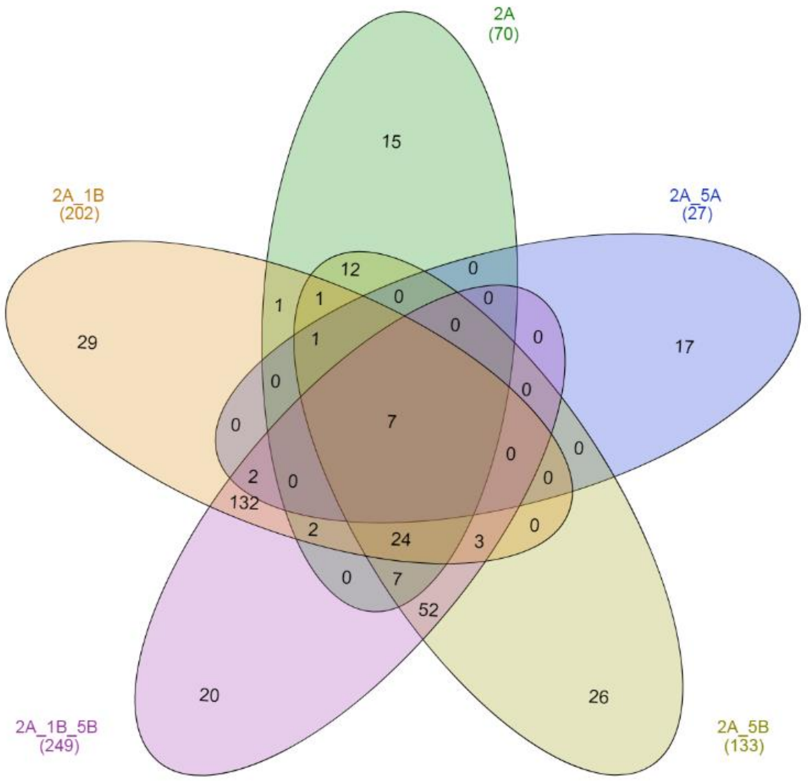

Supplementary file S9. Relationship between differentially expressed genes identified in the five pairwise comparisons of DH lines grouped by the QTL they carried.
